# New secreted toxins and immunity proteins encoded within the Type VI secretion system gene cluster of *Serratia marcescens*

**DOI:** 10.1111/mmi.12028

**Published:** 2012-09-27

**Authors:** Grant English, Katharina Trunk, Vincenzo A Rao, Velupillai Srikannathasan, William N Hunter, Sarah J Coulthurst

**Affiliations:** 1 Division of Molecular Microbiology, College of Life Sciences, University of DundeeDundee, UK; 2 Division of Biological Chemistry and Drug Discovery, College of Life Sciences, University of DundeeDundee, UK

## Abstract

Protein secretion systems are critical to bacterial virulence and interactions with other organisms. The Type VI secretion system (T6SS) is found in many bacterial species and is used to target either eukaryotic cells or competitor bacteria. However, T6SS-secreted proteins have proven surprisingly elusive. Here, we identified two secreted substrates of the antibacterial T6SS from the opportunistic human pathogen, *Serratia marcescens*. Ssp1 and Ssp2, both encoded within the T6SS gene cluster, were confirmed as antibacterial toxins delivered by the T6SS. Four related proteins encoded around the Ssp proteins (‘Rap’ proteins) included two specifically conferring self-resistance (‘immunity’) against T6SS-dependent Ssp1 or Ssp2 toxicity. Biochemical characterization revealed specific, tight binding between cognate Ssp–Rap pairs, forming complexes of 2:2 stoichiometry. The atomic structures of two Rap proteins were solved, revealing a novel helical fold, dependent on a structural disulphide bond, a structural feature consistent with their functional localization. Homologues of the *Serratia* Ssp and Rap proteins are found encoded together within other T6SS gene clusters, thus they represent founder members of new families of T6SS-secreted and cognate immunity proteins. We suggest that Ssp proteins are the original substrates of the *S. marcescens* T6SS, before horizontal acquisition of other T6SS-secreted toxins. Molecular insight has been provided into how pathogens utilize antibacterial T6SSs to overcome competitors and succeed in polymicrobial niches.

## Introduction

Protein secretion systems and their substrates are central to bacterial virulence and interaction with other organisms (Gerlach and Hensel, 2007). Six different secretion systems (Types I–VI) are used by Gram-negative bacteria to transport specific proteins to the exterior of the bacterial cell or further inject them into target cells. The most recently described of these is the Type VI secretion system (T6SS) (Filloux *et al*., 2008). T6SSs are complex multi-protein assemblies that span both bacterial membranes and inject effector proteins directly from the bacterial cytoplasm into target cells (Bonemann *et al*., 2010; Cascales and Cambillau, 2012). T6SSs are encoded by large, variable gene clusters that contain 13 ‘core’ essential components, believed to make up the basic secretion apparatus. Two core proteins, Hcp and VgrG, form the extracellular part of the secretion machinery and depend on a functional T6SS apparatus for their movement to the outside of the bacterial cell (indeed, the presence of Hcp in the secreted fraction has provided a useful assay for basic T6SS assembly and activity; Pukatzki *et al*., 2009). Hcp and VgrG most likely form a needle-like membrane-puncturing device related to the bacteriophage tail spike; this structure is believed to be pushed to the outside of the secreting cell and likely into target cells upon contraction of a tail sheath-like structure (Leiman *et al*., 2009; Bonemann *et al*., 2010; Basler *et al*., 2012). T6SSs occur in many pathogenic bacteria and are implicated in virulence in important pathogens, including *Burkholderia mallei*, *Burkholderia pseudomallei*, *Burkholderia cenocepacia*, *Vibrio cholerae*, *Aeromonas hydrophila*, *Edwardsiella tarda* and *Pseudomonas aeruginosa* (Zheng and Leung, 2007; Cascales, 2008; Jani and Cotter, 2010; de Pace *et al*., 2010; Burtnick *et al*., 2011; Rosales-Reyes *et al*., 2012). In several cases, the action of such ‘anti-eukaryotic’ T6SSs appears to result in disruption of the actin cytoskeleton (Pukatzki *et al*., 2007; Aubert *et al*., 2008; Suarez *et al*., 2010). Exciting recent work has demonstrated that some T6SSs are used to target other bacteria, efficiently killing or inhibiting competitors. This has been reported for T6SSs in *P. aeruginosa*, *Burkholderia thailandensis*, *V. cholerae* and *Serratia marcescens* (Hood *et al*., 2010; MacIntyre *et al*., 2010; Schwarz *et al*., 2010; Murdoch *et al*., 2011). The discovery that certain T6SSs may be ‘antibacterial’ rather than, or in addition to, ‘anti-eukaryotic’ is highly relevant to the competitive fitness and success of pathogens, particularly within polymicrobial infection sites. Such a system could provide the pathogen with a large competitive advantage against other bacteria in the host or the environment, enabling it to proliferate and mount a successful infection.

Identifying the proteins secreted by T6SSs is a priority, as they will be the ‘effectors’ that directly act on target eukaryotic or bacterial cells. A special case of T6-secreted effector is the class of ‘evolved’ VgrG proteins found in a minority of T6SSs which have extra C-terminal effector domains, e.g. the actin cross-linking domain of *V. cholerae* VgrG1 that is translocated into mammalian cells (Pukatzki *et al*., 2007; Jani and Cotter, 2010). However, excluding the structural components VgrG and Hcp, very few ‘true’ T6SS-secreted proteins have been confirmed so far. Best characterized are three effector proteins, antibacterial toxins named Tse1–3, secreted by the antibacterial HSI-1 T6SS of *P. aeruginosa*. Tse1 and 3 are peptidoglycan hydrolases that attack the cell wall of target bacteria (Hood *et al*., 2010; Russell *et al*., 2011). Tse2 is active in the cytoplasm of target cells, where it efficiently induces quiescence (Li *et al*., 2012). All three have adjacently encoded cognate ‘immunity’ proteins (Tsi1–Tsi3) which protect the secreting cell from harming itself or being harmed by its sibling neighbours (Hood *et al*., 2010; Russell *et al*., 2011). Significantly, obvious homologues of the Tse and Tsi proteins are not detectable outside of *P. aeruginosa* (Hood *et al*., 2010). A recent report has also identified a number of candidate T6SS substrates in *B. thailandensis*, one of which was confirmed as a new T6-secreted peptidoglycan amidase (Russell *et al*., 2012).

Opportunistic Gram-negative bacteria cause a large proportion of problematic and antibiotic-resistant hospital-acquired infections. Enterobacteria (especially extended-spectrum β-lactamase producing isolates) are among the leading culprits, including *S. marcescens* (Choi *et al*., 2007; Lockhart *et al*., 2007). We previously reported that *S. marcescens* Db10 possesses a T6SS with potent antibacterial activity (Murdoch *et al*., 2011). How this activity is mediated, in particular the antibacterial effectors secreted by the T6SS, was unknown. Hence, we sought to identify and characterize novel antibacterial effectors secreted by the *S. marcescens* T6SS. We report the identification and characterization of two such effectors, Ssp1 and Ssp2, which are encoded within the T6SS gene cluster and represent novel T6-secreted antibacterial toxins. We have also identified and characterized the Rap proteins, which include the cognate immunity proteins to these toxins. Biochemical analyses demonstrated a tight and specific interaction between secreted and immunity proteins. These secreted toxins and immunity proteins represent two new protein families, co-occurring within T6SS gene clusters of many other organisms. Additionally, determination of high-resolution crystal structures of two members of the Rap protein family revealed that this family possesses a previously undescribed protein fold that is dependent on formation of a disulphide bond.

## Results

### The T6SS gene cluster harbours self-resistance determinants and candidate secreted effectors

**Fig. 1:**
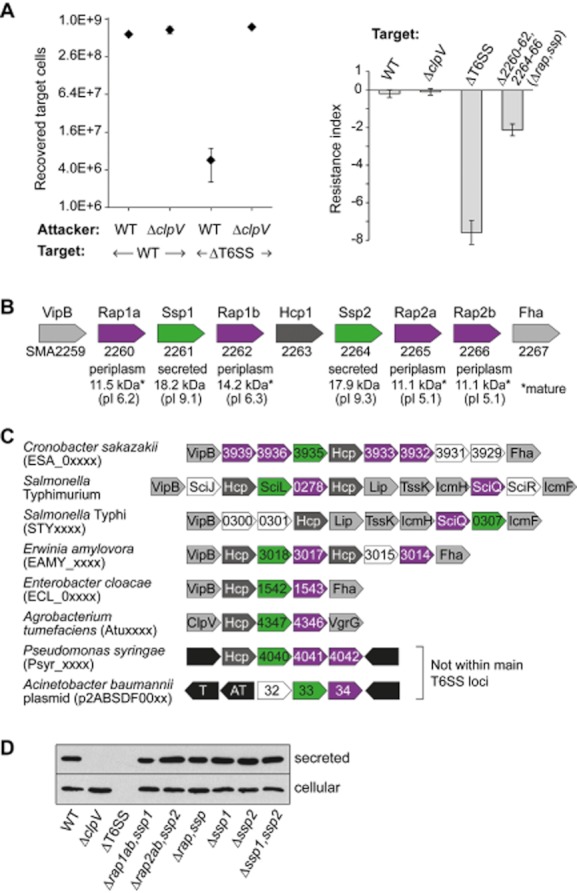
An internal locus in the *S. marcescens* T6SS gene cluster encodes secreted proteins and self-resistance functions. A. T6SS-mediated inhibition of self in the absence of genes within the T6SS gene cluster. Left: number of recovered target cells, either wild type (WT) or ΔT6SS mutant (Δ*SMA2244–2281*), following co-culture (1:1) with the attacking strain, WT or Δ*clpV* (T6SS inactive mutant). Right: resistance index, defined as log_2_[recovery of target in presence of wild type/recovery of target in presence of Δ*clpV*], of wild type Db10, Δ*clpV*, ΔT6SS or a mutant lacking genes *SMA2260–2262* and *SMA2264–2266*. Bars show mean ± SEM (*n* = 4). B. and C. Schematic depiction of loci containing genes encoding the Rap and Ssp genes in *S. marcescens* Db10 (B) and homologues in selected other organisms (C). Rap family proteins are shown in purple, Ssp family proteins in green, conserved T6SS core genes in grey, and Hcp homologues in dark grey. In (B) cellular localization and theoretical mass and pI of the proteins are given beneath the corresponding gene. D. Secretion of Hcp1 by wild type and mutants of *S. marcescens* Db10 as shown by anti-Hcp1 immunoblotting of cellular and secreted fractions. Δ*rap1ab,ssp1* indicates a mutant lacking the *rap1a*, *rap1b* and *ssp1* genes; Δ*rap2ab,ssp2* indicates a mutant lacking the *rap2a*, *rap2b* and *ssp2* genes; Δ*ssp1,ssp2* indicates a mutant lacking the *ssp1* and *ssp2* genes, and Δ*rap,ssp* indicates a mutant lacking all of the *rap* and *ssp* genes.

The T6SS gene cluster of *S. marcescens* Db10, *SMA2244–2281*, contains 38 genes, including many with no known function (Murdoch *et al*., 2011). We speculated that encoded within this cluster might be T6-secreted effectors and/or self-resistance determinants, the latter preventing the T6SS-expressing cell from harming itself or being harmed by its isogenic (sibling) neighbours. In order to determine whether the cluster did indeed contain self-resistance determinants (such as specific immunity proteins analogous to the Tsi proteins), we generated a mutant lacking the entire T6SS gene cluster (ΔT6SS) and examined whether it was fully resistant to the T6SS of the wild type strain. Co-culture of two target strains, wild type Db10 and the ΔT6SS mutant, each with a wild type and a Δ*clpV* attacker, showed that the ΔT6SS mutant had lost resistance to T6SS-mediated inhibition or killing by the wild type strain (Fig. 1A, left). Recovery of ΔT6SS was decreased 100-fold when it was co-cultured with the wild type strain, compared with when the wild type strain was co-cultured with itself. This effect was dependent on a functional T6SS in the attacker as there was no loss of ΔT6SS when it was co-cultured with a Δ*clpV* mutant. The ATPase ClpV is one of the core, structural components of the T6SS and we have shown previously that it is essential for Hcp secretion and T6-mediated antibacterial killing activity of *S. marcescens* Db10 (Murdoch *et al*., 2011). To simplify the analysis of multiple mutants, we defined the ‘resistance index’ of a strain as the difference between recovery when co-cultured with the wild type strain and recovery when co-cultured with the Δ*clpV* mutant, specifically log_2_[recovery vs. wild type/recovery vs. Δ*clpV*]. The wild type or other resistant strain will show no difference and have a resistance index of 0. A target strain with reduced ability to resist harm caused by the T6SS will have a negative resistance index, exemplified by the ΔT6SS mutant with a resistance index of −7.6 (Fig. 1A, right). Self-resistance did not depend on an active T6SS, as a Δ*clpV* mutant had a resistance index of 0. Similarly, mutants in other essential core T6SS components, Δ*lip*, Δ*icmH* and Δ*tssK* (Murdoch *et al*., 2011) also showed no loss of self-resistance (data not shown). Therefore, genes other than those encoding the core conserved T6SS components were implicated in self-resistance. Our attention was particularly caught by a locus in the middle of the gene cluster, where six non-conserved genes, *SMA2260–2262*, *SMA2264–2266*, are flanked by conserved T6SS components. A mutant lacking all of these genes (but maintaining intact *SMA2263*, encoding Hcp1) was also tested and found to have a negative resistance index (Fig. 1A). Hence, one or more of these genes contributes to self-resistance and may encode immunity protein(s).

Closer examination of the proteins encoded by *SMA2260–2262* and *SMA2264–2266* revealed two classes of small proteins (Fig. 1B). SMA2261 and SMA2264 were basic proteins with detectable sequence similarity between them, no discernable cellular localization signals and no predicted function. We hypothesized that they might be secreted substrates, and, given subsequent results, named them Ssp1 and Ssp2 (Secreted small protein). SMA2260, SMA2262, SMA2265 and SMA2266 were proteins with classical Sec-dependent N-terminal signal peptides, identified using SignalP (Petersen *et al*., 2011), and thus predicted to be periplasmic. They also had no predicted function but shared detectable sequence similarity with each other. We hypothesized that they would represent specific immunity proteins or other self-resistance determinants, and named them Rap1a, Rap1b, Rap2a and Rap2b (Resistance associated protein). The genes encoding all these small proteins fall into two sets, either side of *hcp1* and within genes encoding T6SS structural proteins, *vipB* and *fha* (Fig. 1B). Homologues of the Ssp and Rap proteins are encoded within T6SS gene clusters in many other bacterial species (and in at least one case apart from the T6SS) and appear to always co-occur (see representative examples in Fig. 1C). We speculated that the *S. marcescens* Ssp and Rap proteins represented previously unknown combinations of T6-secreted effectors and cognate immunity proteins. Importantly, mutants lacking one, some or all of the small proteins exhibited wild type levels of Hcp secretion (Figs 1D and S1). Thus, none of the Ssp or Rap proteins is required for Hcp secretion, i.e. they play no structural role in the T6SS.

### Ssp1 and Ssp2 are Type VI-secreted effectors

**Fig. 2:**
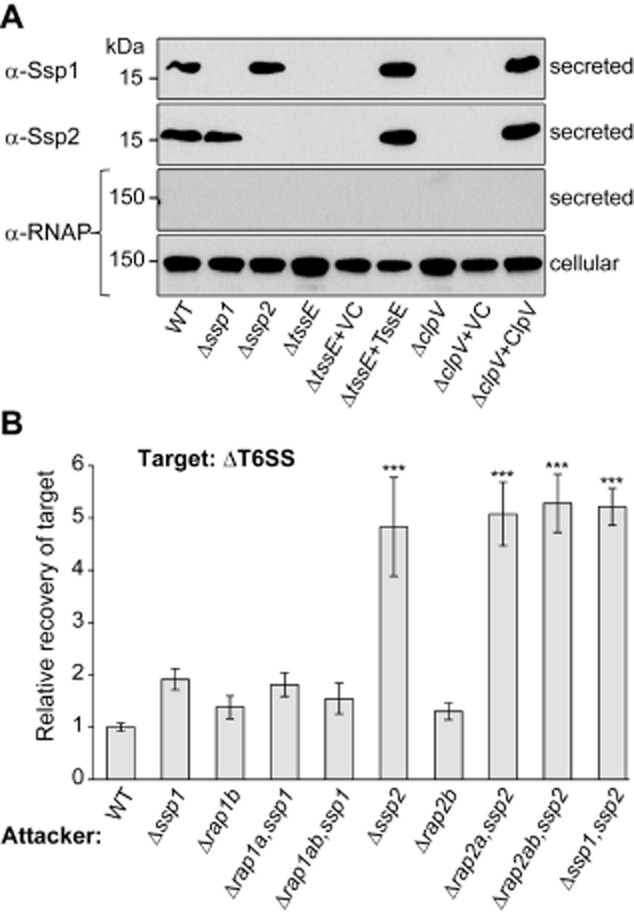
Proteins Ssp1 and Ssp2 are secreted by the Type VI secretion system and self-harm is mediated by Ssp2. A. Immunoblot detection of Ssp1 and Ssp2 in the secreted fraction of the strains indicated using antibodies against Ssp1, Ssp2 or RNAP (lysis control); levels of RNAP in the cellular fraction are also shown. Strains are: wild type *S. marcescens* Db10 (WT); mutants Δ*ssp1*, Δ*ssp2*, Δ*tssE*, Δ*clpV*; mutants carrying vector control plasmids (+VC, pSUPROM) and mutants carrying complementing plasmids (Δ*tssE* + TssE, pSC045; Δ*clpV* + ClpV, pSC039). B. Recovery of the ΔT6SS mutant as the target strain following co-culture with the different attacking strains indicated, expressed relative to recovery of ΔT6SS when co-cultured with wild type Db10. Bars show mean ± SEM (*n* ≥ 4); *** indicates a significant difference compared with the wild type strain (*P* < 0.001).

Ssp1 and Ssp2 were shown to be secreted substrates of the T6SS by immunoblotting secreted fractions from the wild type strain, two T6SS mutants, Δ*clpV* and Δ*tssE*, and the corresponding complemented strains, using specific anti-Ssp1 and anti-Ssp2 antibodies. Both proteins were detected in the culture supernatant, entirely dependent on a functional T6SS (Fig. 2A). Neither protein was detectable in the cellular fraction, implying they were rapidly turned over if not secreted (data not shown). Secretion of Ssp2 was also independent of Ssp1, and vice versa, again confirming they have no structural or accessory role in the secretion machinery (Fig. 2A). In order to establish if the Ssp proteins were antibacterial toxins contributing to the killing or inhibition of the susceptible ΔT6SS mutant by the wild type strain, the recovery of ΔT6SS in the presence of different mutants was determined. Recovery of the ΔT6SS target was increased fivefold with the Δ*ssp2* mutant, or any multiple mutant lacking *ssp2*, as attacker, compared with the wild type attacker (Fig. 2B). In contrast, in this assay, loss of Ssp1 did not cause a statistically significant impairment in killing of ΔT6SS. None of the Rap proteins was required for targeting of ΔT6SS (Fig. 2B).

### Self-resistance against the Ssp1 and Ssp2 toxins is mediated by their cognate Rap partners

**Fig. 3:**
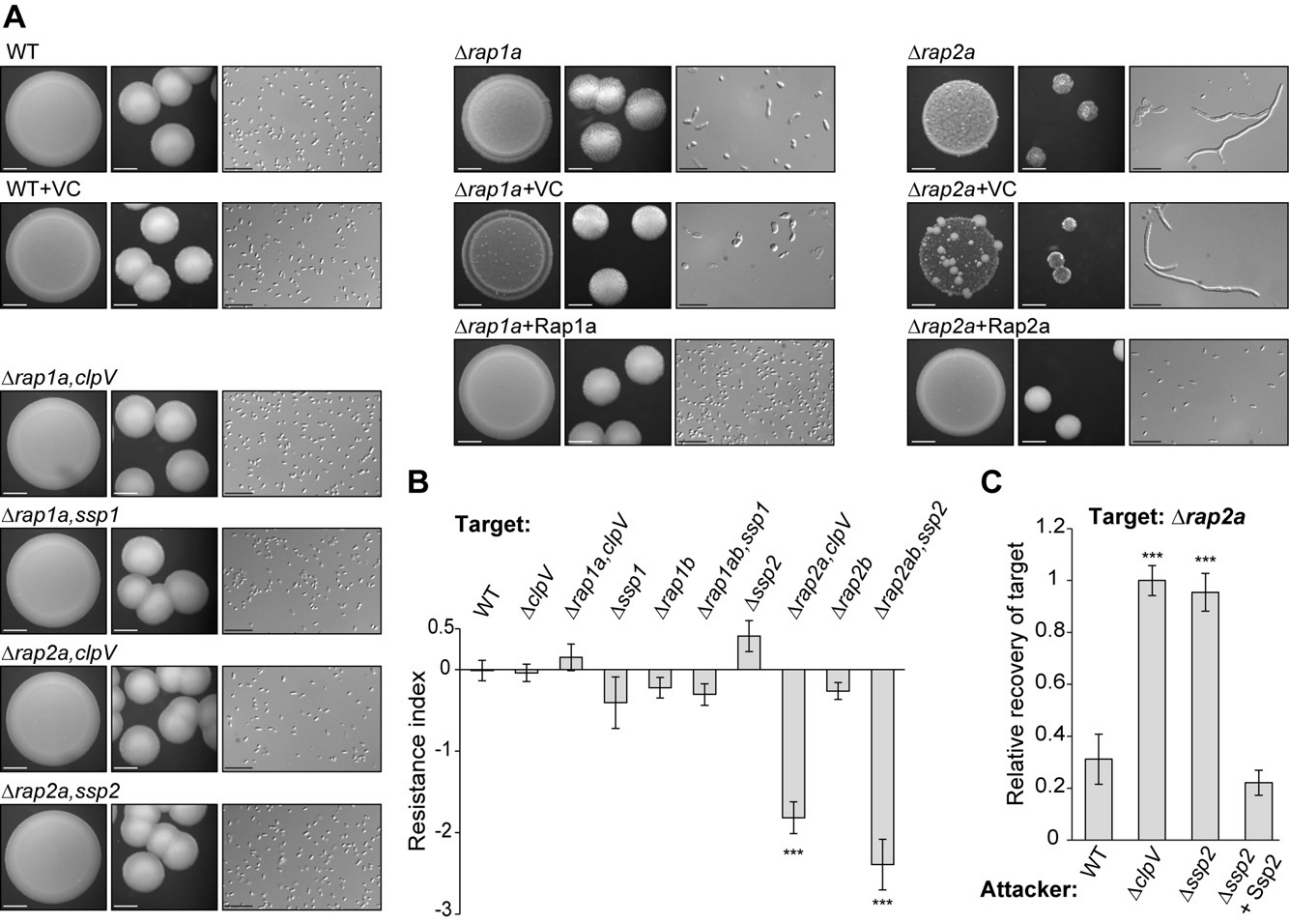
Self-resistance is mediated by specific Rap immunity proteins cognate to the secreted Ssp proteins. A. Phenotypes of wild type *S. marcescens* Db10 (WT) and selected single and double mutants after growth on solid LB media for 24 h. For each strain, representative images of the morphology of a culture spot (left, scale bar 2 mm), single colonies (middle, scale bar 1 mm) and individual cells (right, scale bar 10 μm) are shown. Mutants carrying complementing plasmids are Δ*rap1a* + Rap1a (pSC538) and Δ*rap2a* + Rap2a (pSC542); the vector control plasmid (VC) was pSUPROM. B. Resistance index of wild type Db10 and the deletion mutants indicated as target strains. C. Recovery of a Δ*rap2a* mutant (Δ*rap2a*,Δ*clpV*) as the target strain following co-culture with the different attacking strains indicated, expressed relative to recovery of target when co-cultured with the Δ*clpV* mutant. All strains carry the vector control plasmid pSUPROM, except for Δ*ssp2* + Ssp2, in which the mutant carries the complementing plasmid pSC541. In (B) and (C), bars show mean ± SEM (*n* ≥ 4); *** indicates a significant difference compared with the wild type strain (*P* < 0.001).

In order to assess the contribution of each of the *rap* (and *ssp*) genes to self-resistance, single mutants were constructed. It rapidly became apparent that single mutants in *rap1a* and *rap2a* had severe fitness defects on solid media, both on rich (Fig. 3A) and minimal media (Fig. S2), and particularly Δ*rap2a*. Culture spots and single colonies of each mutant were ‘thinner’, smaller (in the case of Δ*rap2a*), and with altered surface morphology. Examination of single cells showed that the Δ*rap1a* cells appeared bigger and less uniformly shaped than wild type cells and the Δ*rap2a* cells had striking phenotypes, being either markedly distended or highly elongated (Figs 3A and S2). These phenotypes could be complemented by expression of the corresponding gene *in trans* (indeed, the additional stress of the selective antibiotic made the phenotypes of Δ*rap1a* and Δ*rap2a* mutants carrying vector control plasmids even more pronounced; Fig. 3A). In contrast, when Δ*rap1a* was constructed in combination with Δ*ssp1*, or Δ*rap2a* in combination with Δ*ssp2*, the double mutants were readily made and were of normal appearance and fitness on solid media (Figs 3A and S2). These observations are entirely consistent with Rap2a serving as the immunity protein against the Ssp2 toxin and Rap1a being the cognate immunity protein alleviating toxicity mediated by Ssp1. Similarly, when Δ*rap1a* or Δ*rap2a* mutants were constructed in combination with the Δ*clpV* mutation, the double mutants were again apparently healthy on solid media (Figs 3A and S2). Hence, self-toxicity depended on a functional T6SS, implying it was caused by T6SS-mediated injection of the toxin into a susceptible cell by its neighbours (further supported by the observation that Δ*rap1a* and Δ*rap2a* did not display comparable growth defects in liquid culture, where contact-dependent targeting is unlikely to occur efficiently; Fig. S2).

An immunity function for Rap2a was directly demonstrated in co-culture (antibacterial competition assay): target strains containing a Δ*rap2a* mutation all showed a negative resistance index (Fig. 3B; the single Δ*rap2a* mutant was not tested because of its severe sickness). Strains containing a Δ*rap1a* mutation did not have a negative resistance index, consistent with the lack of significant contribution of Ssp1 to self-killing under the conditions of these assays (Fig. 2B). To confirm that Ssp2 was directly and entirely responsible for the inhibitory effect of a wild type attacker strain on Δ*rap2a*, we showed that a Δ*rap2a* target strain is completely resistant to attack by a Δ*ssp2* mutant, with recovery of Δ*rap2a* in the presence of a Δ*ssp2* attacker being the same as its recovery in the presence of a Δ*clpV* attacker (Fig. 3C). Additionally, inhibition of Δ*rap2a* target was restored to wild type levels when the Δ*ssp2* mutant attacker was complemented by expression of Ssp2 *in trans*. The susceptibility of Δ*rap2a* to killing by the wild type strain could also be fully complemented (Fig. S2). Hence, Ssp2 and Rap2a represent a specific, cognate toxin and immunity protein pair.

### Biochemical studies confirm a strong interaction between cognate secreted toxins and immunity proteins

**Fig. 4:**
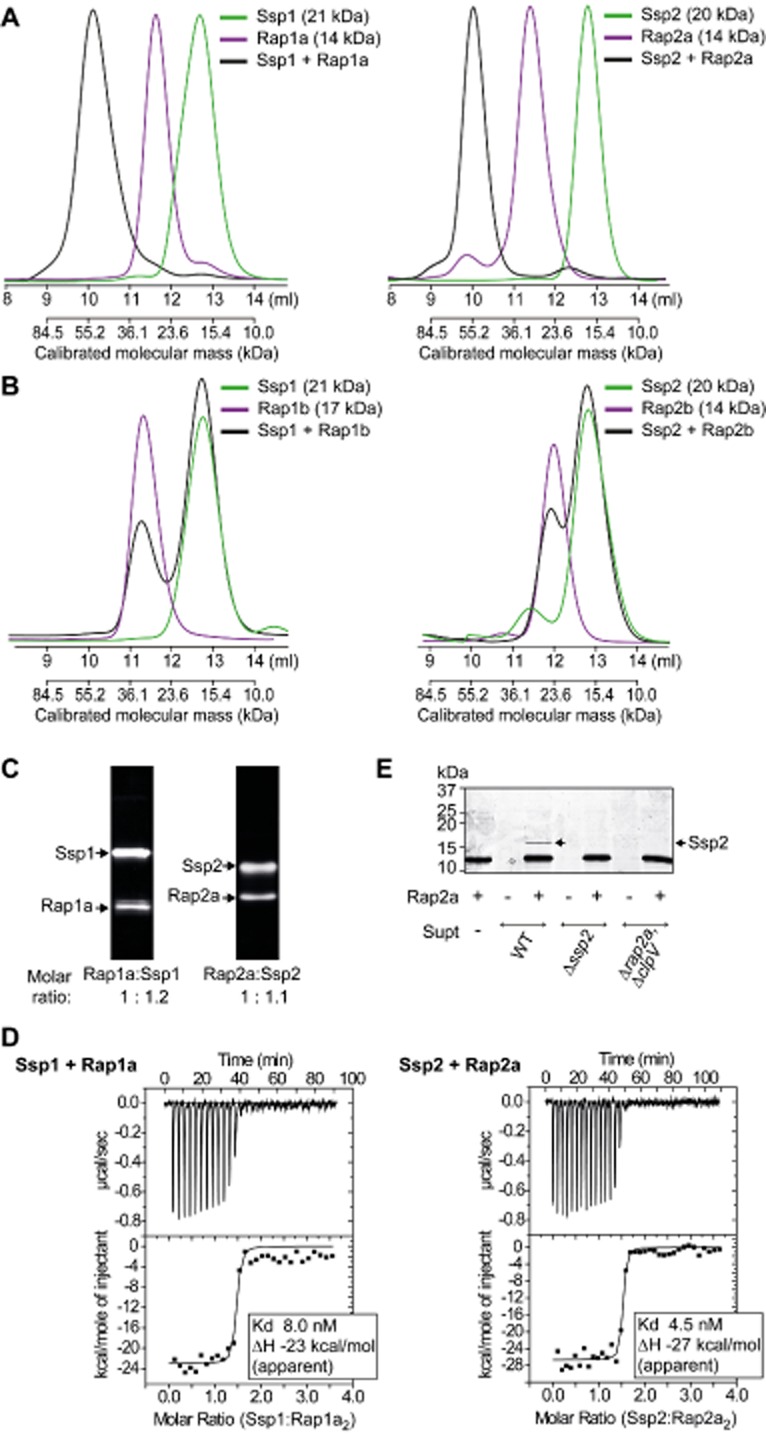
*In vitro* interaction between cognate secreted toxins and immunity proteins. A. and B. SEC analysis of complex formation between the proteins indicated. Ten nanomoles of the protein (or of each protein in the case of mixtures) was separated on a calibrated Superdex 75 10/300 GL column. The theoretical molecular mass of each monomer is given. C. In-gel SYPRO Orange staining and quantification of the relative molar amounts of Rap1a and Ssp1 (left) or Rap2b and Ssp2 (right) in samples from the complex-containing peaks observed in SEC (A). D. ITC analysis of the interaction between Ssp1 and Rap1a (left) and Ssp2 and Rap2a (right). E. Affinity isolation of secreted Ssp2 from culture supernatant using immobilized immunity protein His-Rap2a as bait. Supernatant samples (Supt) were prepared from the strains indicated (WT, wild type). The band indicated by arrowhead was identified as Ssp2 by mass spectrometry.

Each of the Ssp and Rap proteins was overproduced and purified (in the case of the Rap proteins, without their N-terminal signal peptides). Complex formation was demonstrated using size exclusion chromatography (SEC) analysis. All Rap proteins were dimeric in solution, whereas Ssp1 and Ssp2 were monomeric (Fig. 4A and B). When equimolar amounts of Ssp1 and Rap1a were mixed, all of the protein was detected in a higher molecular weight complex (Fig. 4A) and the same was also observed on mixing Ssp2 and Rap2a (Fig. 4A). In contrast, no complex formation between Ssp1 and Rap1b or between Ssp2 and Rap2b, no additional three-way complexes and no ‘cross’ interactions between Ssp2 and Rap1a or between Ssp1 and Rap2a were observed (Figs 4B and S3). As mixing equimolar amounts of Ssp1 and Rap1a resulted in detection of only the complexed species, with no unbound form of either protein detectable, this implied a 1:1 molar complex. The 1:1 molar stoichiometry of the Rap1a–Ssp1 complex was confirmed by quantitative analysis of the composition of the higher molecular weight peak using in-gel SYPRO Orange staining (Rickman *et al*., 2004) (Fig. 4C). Given the dimeric nature of Rap1a and an apparent complex mass by SEC of around 55 kDa [predicted M_w_ of 1:1 molar complexes are 34.6 kDa (1:1), 69.2 kDa (2:2), 104 kDa (3:3) or 138 kDa (4:4)], this is most consistent with a heterotetrameric Rap1a_2_–Ssp1_2_ complex. Similar logic supported a Rap2a_2_–Ssp2_2_ complex (Fig. 4A and C). The SEC analyses suggested stable complexes were formed between the cognate Ssp and Rap proteins. Isothermal titration calorimetry (ITC) analysis of complex formation between Ssp1 and the Rap1a dimer and between Ssp2 and the Rap2a dimer (Fig. 4D) showed that, in both cases, binding was clearly exothermic and tight, at least in the low nanomolar K_d_ range. Although apparent K_d_ values of 4–8 nM were obtained, this is approaching the limit of accuracy for conventional ITC (Wiseman *et al*., 1989). Finally, we took advantage of the tight Rap–Ssp interactions to utilize immobilized Rap2a and Rap1a to affinity-purify secreted Ssp2 or Ssp1, respectively, from culture supernatant (Figs 4E and S3). Mass spectrometry not only confirmed the identity of Ssp1 and Ssp2 but also showed that Ssp2 is not processed on secretion, as almost complete sequence coverage revealed intact N- and C-termini (Fig. S3). For Ssp1, we observed an intact C-terminus but the sequence of the very N-terminus corresponds to several tryptic peptides too small to be detectable by standard mass spectrometry, so we were unable to definitively confirm lack of processing at this end (data not shown).

### The secreted small proteins, Ssp1 and Ssp2, exert distinct harmful effects when targeted to the periplasm in *Escherichia coli* and are neutralized by coexpression of the cognate immunity protein

**Fig. 5:**
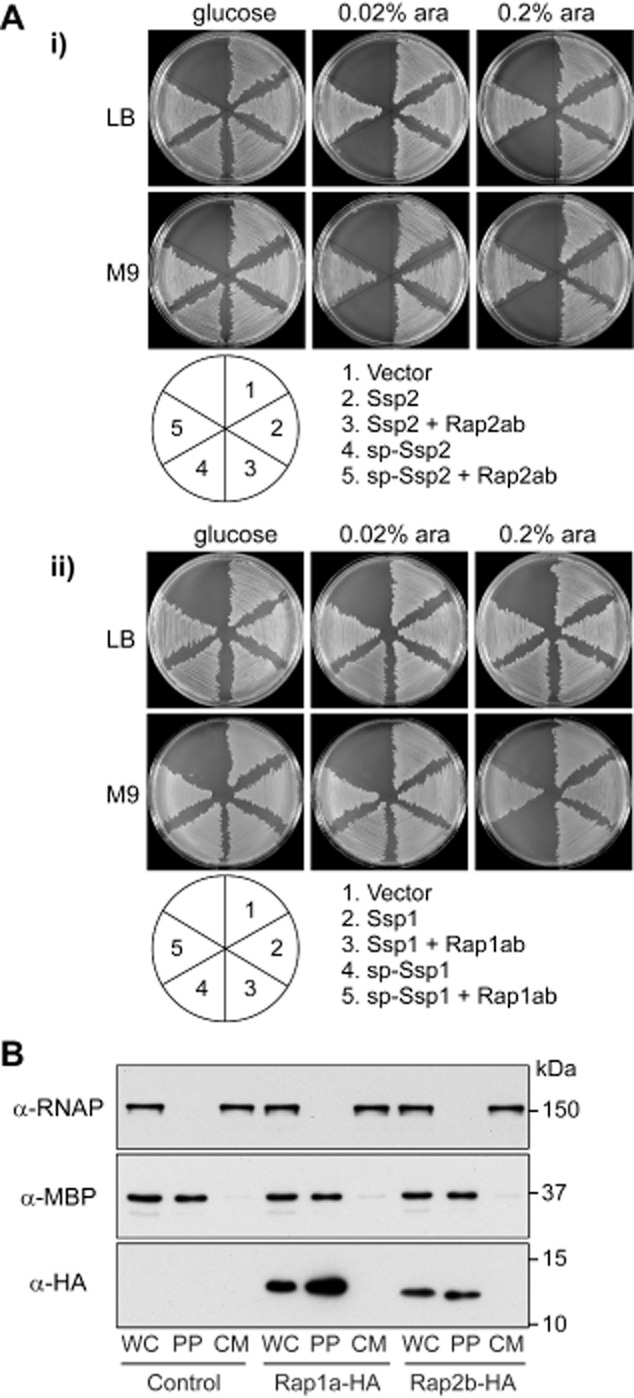
Differential periplasmic toxicity of Ssp1 and Ssp2 and periplasmic localization of Rap proteins. A. Growth of *E. coli* MG1655 transformed with (i) plasmids expressing Ssp2 (pSC133), Ssp2 + Rap2a + Rap2b (pSC134), OmpA_sp_-Ssp2 (pSC138) or OmpA_sp_-Ssp2 + Rap2a + Rap2b (pSC144) from an arabinose-inducible promoter, or with the empty vector (vector, pBAD18-Kn), on LB or M9 media containing 0.2% glucose, 0.02% arabinose or 0.2% arabinose; or (ii) the equivalent analysis using plasmids expressing Ssp1 (pSC151), Ssp1 + Rap1a + Rap1b (pSC159), OmpA_sp_-Ssp1 (pSC152) or OmpA_sp_-Ssp1 + Rap1a + Rap1b (pSC160). B. Localization of RNAP (RNA polymerase, cytoplasmic marker protein), MBP (maltose binding protein, periplasmic marker protein) and HA-tagged Rap proteins. Wild type *S. marcescens* Db10 expressing Rap1a-HA (pSC538), Rap2b (pSC543) or the vector control (pSUPROM) was subjected to fractionation (WC, whole cell; PP, periplasm; CM, cytosol + membranes), followed by immunoblotting with anti-RNAP, anti-MBP or anti-HA antibodies as indicated.

In order to confirm the antibacterial toxin function of Ssp1 and Ssp2 and establish in which cellular compartment they exerted their effect, each protein was produced in *E. coli*, either in the cytoplasm or artificially targeted to the periplasm (by the N-terminal fusion of the Sec-dependent OmpA signal peptide: sp-Ssp2 or sp-Ssp1). The presence of Ssp2 in the periplasm, but not the cytoplasm, prevented growth of *E. coli* on LB and minimal media; this toxicity was alleviated by the co-production of Rap2ab (Fig. 5A). Periplasmic Ssp1 was also toxic and its effect alleviated by co-production of Rap1ab (Fig. 5A). However Ssp1 toxicity was only observed at higher expression levels and on minimal media. The periplasmic localization of the Rap proteins in *S. marcescens* was confirmed by fractionation and immunoblotting of epitope-tagged versions of the protein (Fig. 5B, data not shown).

Next, we also determined whether Ssp2 and/or Ssp1 were essential for the observed antibacterial activity of the *S. marcescens* T6SS against other bacterial species (Murdoch *et al*., 2011). Neither protein was required for T6SS-dependent killing of other bacteria (Fig. S4). This implies redundancy of effector function, in other words that other T6SS-secreted toxins are still able to cause killing in the absence of Ssp1 and Ssp2.

### The Rap family of proteins display a new fold

**Fig. 6:**
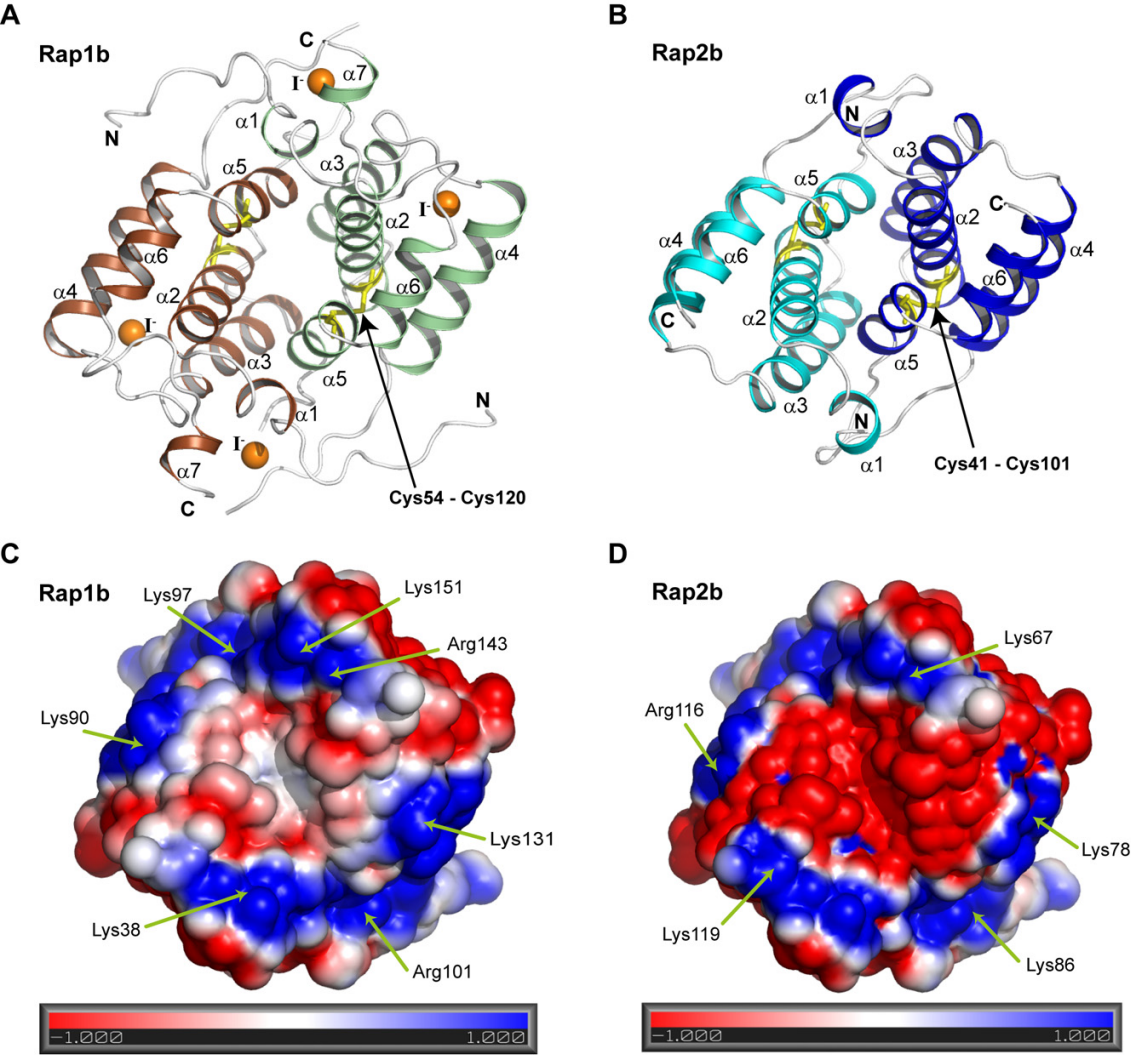
Structural features of Rap1b and Rap2b dimers. The dimers are oriented to provide a view down their twofold axis of symmetry. A. Ribbon diagram with helices of one Rap1b subunit coloured green, and the symmetry related molecule bronze. Elements of secondary structure, the N- and C-terminal residues are labelled and I^−^ ions are depicted as orange spheres. The disulphide bond is shown as yellow sticks and labelled. B. Ribbon diagram of Rap2b with helices of one subunit blue, and the partner cyan. C. and D. Electrostatic surface representation of Rap1b and Rap2b dimers in the same orientation as in (A) and (B). The electrostatic charge is contoured at 1 kT/e and −1 kT/e; negative (acidic) charge is red, positive (basic) blue. Residues that contribute to the basic patches are identified.

Bioinformatic analysis of the Rap protein sequences predicted similarities between them, despite relatively low sequence conservation, e.g. mature Rap1b and Rap2b share around 20% identity, as do mature Rap2a and Rap2b. All four Rap proteins appeared to be highly acidic, to carry an N-terminal periplasmic targeting signal sequence of about 24 residues, and were predicted to have similar α-helical structures (using *PSIPRED*). In addition, a cysteine pairing appeared to be conserved. Intriguingly, no structural relative of the Rap proteins could be identified; hence, we sought to obtain three-dimensional models using single crystal X-ray diffraction. Soluble recombinant Rap proteins were only obtained in high yield from *E. coli* Rosetta-gami (DE3), a strain engineered to support disulphide bond formation, and were dimeric in solution (Fig. 4). Well-ordered crystals of Rap1b and Rap2b were obtained and the structures were elucidated at 1.9 Å and 2.0 Å resolution respectively (Fig. 6, Table 1). A search for structural relatives using *PDBeFold* (pdbe.org/fold) and *ProFunc* (Laskowski *et al*., 2005) was performed using monomers and dimers of Rap1b and Rap2b as templates. The only matches were to short segments of α-helices with Z-scores (< 3) indicating low statistical significance. The lack of any convincing structural relationships indicates that the fold observed in both Rap1b and Rap2b is previously uncharacterized.

**Table 1:** Crystallographic statistics for the Rap1b and Rap2b structures

	Rap1b	Rap2b
Space group	*P*3_1_21	*P*222_1_
*a*, *b*, *c* (Å)	77.9, 77.9, 50.6	48.1, 57.0, 122.4
Resolution^a^ (Å)	67.5–1.88 (1.98–1.88)	44.78–2.0 (2.1–2.0)
No. reflections recorded	162 739 (21 729)	271 849 (36 384)
Unique reflections	14 813 (2130)	23 666 (3390)
Completeness (%)	100.0	100.0
Multiplicity/<*I/σI*>	11.0 (10.2)/26.6 (5.9)	11.5 (10.7)/25.7 (10.0)
Anomalous completeness (%)	100.0	100.0
Anomalous redundancy	5.6 (5.1)	6.0 (5.5)
Wilson *B* (Å^2^)	28.3	20.6
Residues/waters/ligands	119/117/7	392/137/–
*R_merge_*^b^ (%)	5.3 (38.3)	8.5 (35.0)
*R_work_*^c^, *R_free_*^d^ (%)	18.3/22.1	18.9/24.8
Ave. *B*-factor (Å^2^)		
Chain A, B, C, D	21.1	10.6, 9.8, 7.8, 8.9
Waters, iodides, ethylene glycol	42.3, 49.4, 55.7	14.6
Cruickshank DPI^e^ (Å)	0.1	0.2
Ramachandran plot		
Most favoured	118 residues	381
Additional allowed	1	10
Outliers	0	Gln74
R.m.s.d. on ideal values		
Bond lengths (Å) angles (°)	0.02/1.51	0.02/1.79

a. Values in parentheses refer to the highest resolution shell.

b. *R_merge_* = Σ*_hkl_*Σ*_i_|*I*_i_*(*hkl*) − < I(*hkl*) > *|/*Σ*_hkl_*Σ*_i_* I*_i_*(*hkl*), where I*_i_*(*hkl*) is the intensity of the *i*th measurement of reflection *hkl* and <I(*hkl*)> is the mean value of I*_i_*(*hkl*) for all *i* measurements.

c. *R_work_* = Σ*_hkl_*‖*F_o_*| − |*F_c_*‖/Σ|*F_o_*|, where *F_o_* is the observed structure factor and *F_c_* is the calculated structure factor.

d. *R_free_* is the same as *R_cryst_* except calculated with a subset, 5%, of data that are excluded from the refinement calculations.

e. Diffraction Precision Index (Cruickshank, 1999).

The PDB accession codes are 4AX2 (Rap1b) and 4B6I (Rap2b).

The asymmetric unit of the Rap1b structure consists of a single subunit and crystallographic symmetry generates the dimer. Rap2b has two dimers, formed by subunits A : B and C : D, in the asymmetric unit. The four subunits are similar, with the root-mean-square deviation (r.m.s.d.) between superimposed Cα atoms ranging from 0.5 Å (subunit B and D) to 0.7 Å (subunits A and C). The two proteins display obvious similarities in terms of secondary, tertiary and quaternary structure (Figs 6 and S5). An overlay of Rap1b and Rap2b subunits matches 71 Cα atoms with a r.m.s.d. of 1.4 Å. Of note is the conservation of the disulphide bond, a key structural feature, likely critical to the stable folding of the subunit and subsequent dimerization of the Rap proteins. The Rap subunit is constructed around a helical bundle of five helices (α2–α6). A disulphide linkage (Cys54–Cys120 in Rap1b, Cys41–Cys101 in Rap2b) tethers α2 and α5 together. This association in turn supports formation of a hydrophobic core that is primarily aliphatic with residues contributed from α2, α4, α5 and α6. Three helices (α2, α3, α5) form a concave surface on one side of the monomer. A series of hydrogen bonding interactions also helps to stabilize the arrangement of α2 and α4. By virtue of being longer than Rap2b, Rap1b has an extension of five residues at the N-terminus and 13 at the C-terminus, the latter of which form a short helix α7. These segments of Rap1b are on the surface of the molecules at opposite ends of the dimer (Fig. 6).

**Fig. 7:**
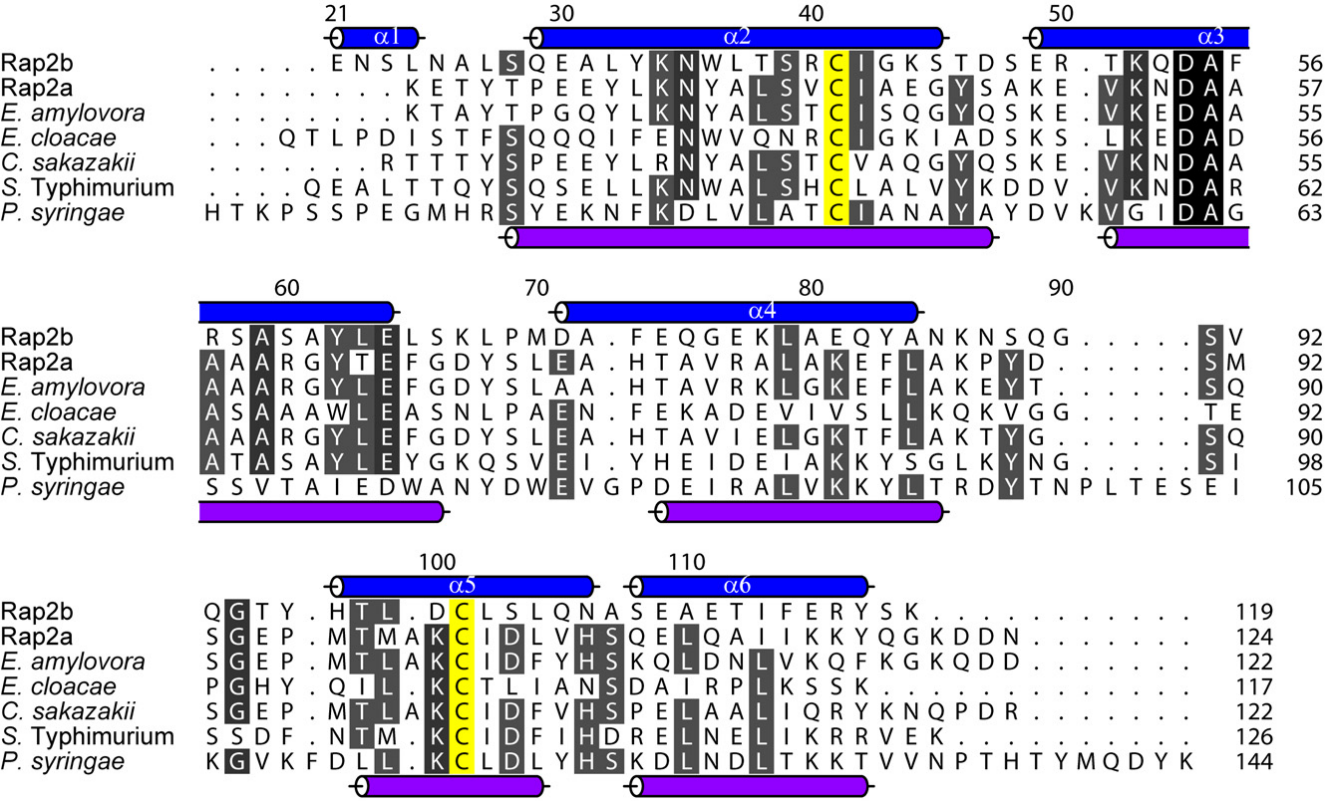
Conservation of the Rap protein fold. Sequence alignment of *S. marcescens* Rap2b and Rap2a with homologous proteins from *Erwinia amylovora* (GenBank CBA22869.1), *Enterobacter cloacae* (NCBI Reference Sequence YP_003612051.1), *Cronobacter sakazakii* (NCBI Reference Sequence YP_001439955.1), *Salmonella* Typhimurium (NCBI Reference Sequence NP_459276.1) and *Pseudomonas syringae* (NCBI Reference Sequence YP_237109.1). The secondary structure of Rap2b (blue cylinders) and the predicted secondary structure of the *P. syringae* protein (purple cylinders) are shown. All proteins had similar predicted secondary structures (*PSIPRED*); the one shown is representative. Cysteine residues involved in disulphide bond formation are coloured yellow. Alignment was generated using *T-Coffee* and annotated using *ALINE*, using the mature proteins (i.e. without N-terminal signal peptides; numbering refers to the full-length proteins).

The Rap proteins display an extensive dimerization interface (Fig. 6), consistent with the observation that each Rap protein exists as a stable dimer in solution (Fig. 4A and B). The Rap1b dimer uses almost 24% of the accessible surface area of a subunit in formation of the dimer. In the case of Rap2b the value is 20%. Such percentages are indicative of highly stable oligomers (Krissinel and Henrick, 2007). The dimer is stabilized by extensive van der Vaals interactions primarily involving aliphatic residues, in conjunction with hydrogen bonding interactions and solvent mediated bridging associations. The most important contributions are from side-chains on the concave surface, formed by α2, α3 and α5, interacting with the partner across the molecular twofold axis of symmetry. Additional interactions involve a self-association of the loop that links α2 and α3, together with the N-terminus α1 and α5. Helices α2 and α5 are thus not only critical for the fold of the Rap subunits but also to creating a suitable interface that leads to a highly stable dimer. This observation, together with the conserved disulphide bond (Fig. 7), ties in with the localization of Rap proteins to the oxidative environment of the periplasm where correct folding to support anti-toxin activity must occur.

Importantly, sequence analysis and secondary structure predictions for the other Rap proteins, in *S. marcescens* and other organisms, suggest strongly that the novel fold revealed by our crystallographic analyses is generic for this entire family of proteins (Fig. 7). Not only are the main α-helices observed in both crystal structures closely mimicked by the structural predictions of the other members of the family, the Cys residues contributing to the structural disulphide bond are also conserved. Additionally, Rap2b shares slightly higher sequence identity with Rap2a (23% identity for the mature proteins) than it does with Rap1b (20% identity). Given the similarity of the Rap1b and 2b crystal structures, discussed above (Fig. S5), then this provides confidence that Rap2a shares this new fold and indeed that the Rap fold is a defining characteristic of this protein family, many members of which are likely to represent T6SS immunity proteins.

## Discussion

In this study we have identified two proteins, Ssp1 and Ssp2, as true (non-structural) secreted substrates of the *S. marcescens* T6SS and confirmed them as new T6SS-dependent antibacterial toxins. We have also demonstrated, genetically and biochemically, that the highly specific, cognate periplasmic immunity proteins, Rap1a and Rap2a, efficiently neutralize the effect of the Ssp proteins. The atomic structures of two other, related Rap proteins reveal that the immunity proteins should exhibit a novel protein fold likely only attained when they are present in the periplasm.

Our examination of Ssp1 and Ssp2 suggested that they represented novel antibacterial toxins, containing a domain of unknown function, DUF4285, present in prokaryotic and eukaryotic proteins. We were unable to detect significant sequence similarity with well-known peptidoglycan hydrolases and the structure prediction program Phyre2 (Kelley and Sternberg, 2009) was unable to assign them any peptidoglycan hydrolase-like structure. Nevertheless, as they are clearly periplasmic-acting toxins (Fig. 5) and non-resistant mutants showed apparent lytic and/or division defects (Figs 3A and S2), a cell wall targeting function seemed most likely. This idea has very recently been strongly supported by the observation that related proteins exhibit peptidoglycan amidase activity *in vitro* (see below; Russell *et al*., 2012). Importantly, through analysing secreted Ssp2 isolated from culture supernatant, we have also shown for the first time that T6-secreted effectors are not processed at either terminus during secretion. It seems clear that Ssp1 and Ssp2 are not the only effector proteins secreted by the *S. marcescens* T6SS. First, Ssp mutants are still able to kill *Pseudomonas fluorescens* as effectively as wild type Db10 (Fig. S4), implying that other toxins secreted in their absence are sufficient to maintain efficient antibacterial killing. Additionally, the susceptibility of the ΔT6SS mutant to self-targeting by the wild type strain is greater than that of the Δ*rap,ssp* mutant (Fig. 1A), implying additional immunity proteins and thus cognate secreted toxins within the T6SS gene cluster. Moreover, the magnitude of killing of the ΔT6SS mutant by wild type Db10 is less than that observed during T6-dependent killing of other organisms (Murdoch *et al*., 2011; Fig. S4), suggesting that additional secreted toxins (and cognate immunity proteins) are encoded elsewhere in the genome. Indeed, our unpublished work has identified four other, unrelated, candidate substrates of the *Serratia* T6SS. Hence, we believe that *S. marcescens* uses a species-specific arsenal of secreted toxins to produce the potent and efficient targeting of a variety of competitor bacteria observed (Murdoch *et al*., 2011).

Our work provides strong functional evidence for a new family of related T6SS substrates (Ssp1 and Ssp2 homologues) and family of related immunity proteins (Rap homologues) found in many different bacterial species and generally encoded within a main T6SS gene cluster of that organism. Ssp- and Rap-like proteins are found associated with a subset of T6SSs (some, but not all, closely related to the *S. marcescens* T6SS), but whether these T6SSs all exhibit antibacterial activity remains to be determined. While this report was in preparation, a bioinformatic study identified four disparate families of predicted T6-secreted peptidoglycan amidases, with Tse1 of *P. aeruginosa* being a member of ‘Family 1’ (Russell *et al*., 2012). Entirely consistent with our data, Ssp-like proteins were recognized as one of these families, ‘Family 4’. A related family of proposed cognate immunity proteins identified as co-occurring with all Ssp/Family 4 proteins is of course the Rap family proteins. Thus, in the nomenclature proposed by Russell *et al*., Ssp1 and Ssp2 could be classified as Tae4.1^SM^ and Tae4.2^SM^, and Rap1a and Rap2a as Tai4.1a^SM^ and Tai4.2a^SM^. Unlike Families 1–3, the Family 4/Ssp proteins are almost unrecognizable as peptidoglycan amidases at a sequence and structure prediction level. Nevertheless, the purified Ssp homologue STM0277 from *Salmonella enterica* serovar Typhimurium was able to hydrolyse peptidoglycan cross-links at the D-Glu-*m*DAP bond of the acceptor stem (Russell *et al*., 2012). Additionally, artificial expression and targeting of STM0277 to the periplasm was shown to be toxic to *E. coli*, with rescue by coexpression of the Rap homologue STM0278. However, studies were not conducted to show that STM0277 is a T6SS substrate or that it plays a role in T6-mediated antibacterial activity, neither was a role for STM0278 in self-resistance in the native, T6-elaborating organism investigated. In contrast, we have provided the comprehensive genetic, *in vivo* and biochemical data necessary to confirm that Ssp/Family 4 proteins are indeed a novel family of T6-secreted antibacterial toxins and moreover that the Rap family contains the cognate immunity proteins. Interestingly, it was recently reported that a mutant in the above Rap2a homologue in *S.* Typhimurium, STM0278, had a defect in replication in macrophages (Mulder *et al*., 2012). The reason behind this is not clear, but it may reflect the fact that this mutant has reduced fitness due to self-toxicity, just as we have shown for the Δ*rap2a* mutant. It should also be noted that Ssp–Rap pairs are not always found associated with T6SS genes. A particularly interesting example is the location of such a pair almost adjacent to genes encoding a classical RelE–RelB toxin–antitoxin pair (T-AT; Yamaguchi and Inouye, 2011) on a plasmid in *Acinetobacter baumannii* (Fig. 1). It is tempting to speculate that an original source of T6 toxin/resistance pairs is from plasmid T-AT systems.

The work of Russell *et al*. (2012) combined with our demonstration that Ssp1 and Ssp2 function as periplasmic-acting toxins is highly consistent with these proteins having a peptidoglycan amidase enzymatic activity. Of particular note, conserved Cys and His residues predicted by Russell *et al*. to represent the catalytic amino acids mediating peptidoglycan amide bond hydrolysis can be readily identified in Ssp1 and Ssp2. These are Cys50 (NTCAVRMS) and His133 (GHIDLIEP) in Ssp1, and Cys50 (NACAIRMS) and His131 in Ssp2 (GHATLWNG), with the equivalent, conserved regions in STM0277 being (NACPIRMS and GHVTLWNG). However, crucially, our data on the Ssp and Rap proteins in the context of T6SS-mediated attack and defence *in vivo* reveal that the situation is more subtle than this. In particular, Ssp1 and Ssp2 are clearly not redundant, despite having the same postulated enzymatic function. Rather, they have distinct activities or roles, with Ssp2 apparently more potent. In particular, we noted that the Ssp-dependent morphological phenotypes of the Δ*rap1a* and Δ*rap2a* mutants are different (Figs 3A and S2), that toxicity in the *E. coli* periplasm is only medium-dependent for Ssp1 (Fig. 5), and that the two may be relevant in different biological contexts (e.g. Ssp1 does not significantly contribute to self-targeting under the conditions of our standard assay, yet is clearly required for the self-toxicity observed in a Δ*rap1a* mutant). This specialization is consistent with a clear specificity for only the cognate Ssp–Rap partner, as we observed. In the native context, it is clear that none of the other three Rap proteins can confer cross-resistance to Ssp2 in the absence of Rap2a (nor did Ssp2 interact with Rap1a biochemically). The molecular basis for the difference between Ssp1 and Ssp2, which share 24% sequence identity, is not yet clear and will require further study, including determination of *in vitro* enzymatic activity and atomic structures.

The *S. marcescens* Rap proteins represent founder members of a new bacterial protein family, members of which represent immunity proteins for T6-secreted toxins, as exemplified by Rap1a and Rap2a. We have determined the structures of two members of this family, Rap1b and Rap2b, revealing a new protein fold. Given the sequence homology, conservation of key residues and shared predicted secondary structure throughout all members of the family (Fig. 7; Russell *et al*., 2012), this fold appears to be shared across members from different organisms. A conserved disulphide bond and the observed stable dimerization interface, together with canonical N-terminal signal sequences, are consistent with a periplasmic localization for all Rap family proteins. To date, the structures of two other T6 immunity proteins have been solved. The first is the cytoplasmic Tsi2 protein (Li *et al*., 2012; Zou *et al*., 2012). Like the Rap proteins, Tsi2 exhibits a helical fold, is acidic and exists as a stable dimer in solution. However, the structures of Tsi2 and the Rap proteins are unrelated. Second, and very recent, is the structure of the periplasmic Tsi1 protein, complexed with the secreted peptidoglycan hydrolase effector, Tse1 (Ding *et al*., 2012). Tse1 possesses a strikingly accessible active site, facilitating its promiscuous and toxic peptidoglycan amidase activity (Chou *et al*., #b^1001^). Tsi1 binds to Tse1 in a 1:1 complex, occluding the substrate-binding site of Tse1 in order to neutralize its activity (Ding *et al*., 2012). Critically, although the Rap1a/Rap2a proteins and Tsi1 both mediate resistance to peptidoglycan hydrolase toxins, their structures now appear to be entirely unrelated. Tsi1 exhibits an all β fold, related to a classical β-propeller, whereas Rap family proteins exhibit a novel helical fold. This indicates that the mechanisms by which immunity proteins confer resistance may be divergent even among those with effectors of similar function. Rap1a and Rap2a have an obvious immunity phenotype specific to their cognate secreted toxins, Ssp1 and Ssp2. However, the role of Rap1b and Rap2b is not yet known. While they do not appear to play a role in self-resistance, they may play a role in resistance towards closely related bacteria secreting similar toxins. It is worth noting that other organisms also possess multiple Rap family proteins for a given Ssp family protein (Fig. 1C and data not shown); therefore, whatever the function of Rap1b and 2b turns out to be, it may not be unique to *Serratia*.

Complementing our genetic and phenotypic demonstration of the cognate toxin-immunity function of Ssp1–Rap1a and Ssp2–Rap2a, we report detailed biochemical characterization of the interactions between these purified T6-secreted toxins and immunity proteins. For efficient self-protection, these interactions should be tight and highly specific, as was observed. Formation of Ssp1–Rap1a and Ssp2–Rap2a complexes is exothermic, with low nanomolar K_d_, and with a stoichiometry of 2:2. A binding affinity of this order agrees well with the K_d_ of 3 nM reported for the Tsi1–Tse1 interaction (Ding *et al*., 2012); however, the Tsi1–Tse1 complex has a stoichiometry of 1:1, again highlighting significant differences between different pairs of toxin-immunity proteins. The structure of a Ssp–Rap complex and arrangement of the subunits has yet to be determined, although it is likely that the two Ssp proteins bind to the same part of each Rap monomer, exploiting the twofold symmetry. Li *et al*. suggest that Tse2 interacts with an acidic patch on Tsi2 distal to the dimer interface (Li *et al*., 2012). They also note that, like many toxin–antitoxin pairs, the resistance proteins Tsi1–3 are more acidic than the toxin proteins Tse1–3. This pattern is strikingly followed with the Ssp (theoretical pI 9) and Rap (theoretical pI 5–6) proteins. Nevertheless, while the highly acidic Rap proteins bind their cognate Ssp partners, which are noticeably basic, with high affinity, the story is not as simple as charge complementarity. Surprisingly for proteins with such low pI values, the striking surface feature conserved in Rap1b and Rap2b structures is a crescent-shaped basic patch on either side of the dimer (Fig. 6C and D). It is possible that such a basic crescent, if present on toxin-binding Rap proteins, may contribute to orientation of the partner during binding; conversely, it might contribute to the lack of Ssp binding by Rap1b and Rap2b. Additionally, all four *S. marcescens* Rap proteins are acidic and both Ssp proteins basic, yet only two, highly specific interactions are observed (Rap1a–Ssp1 and Rap2a–Ssp2). While charge complementarity would be expected to be important for specific complex formation, it is likely that shape considerations play an equally important role. The structure of a toxin-immunity protein complex will be necessary to delineate the molecular features that govern specific Rap–Ssp association. We are working towards that goal.

**Fig. 8:**
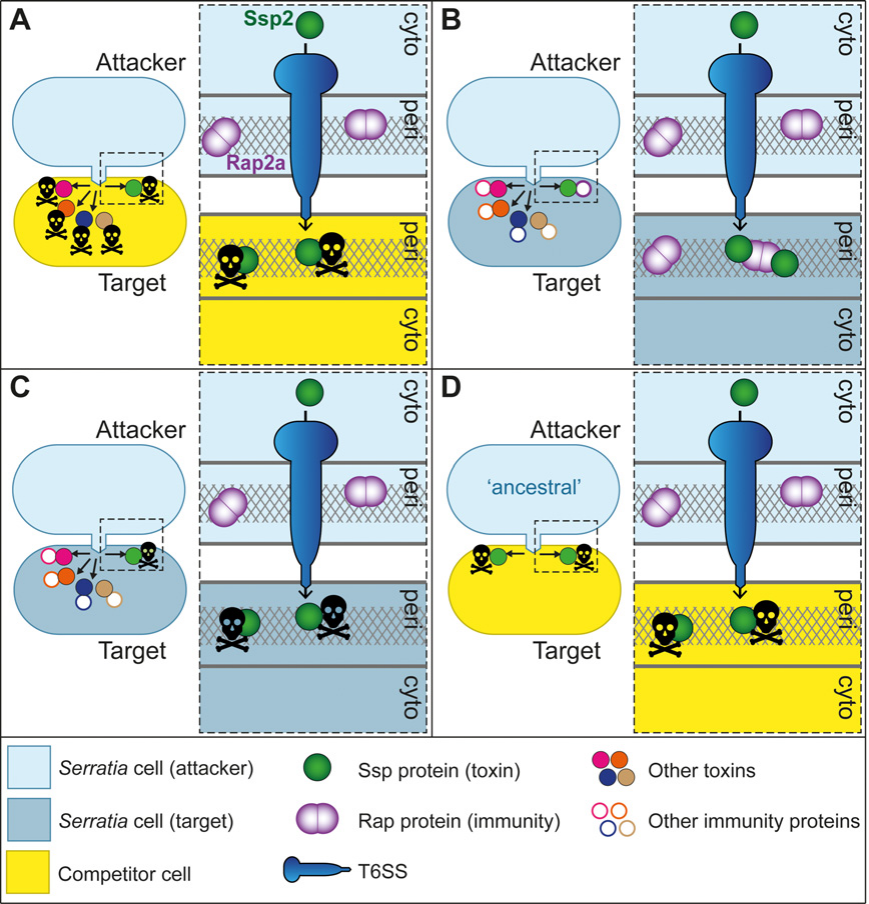
Model for action and context of Ssp and Rap proteins. A. Wild type *S. marcescens* Db10 uses its T6SS to inject multiple different antibacterial toxins (solid circles), including Ssp2 (green), into a susceptible target competitor cell, cumulatively causing a rapid death. Ssp2 attacks the cell wall in the periplasm (peri); other toxins are likely to attack targets in the cytoplasm (cyto). B. Another wild type cell is resistant to T6SS attack by its neighbour because of the presence of cognate immunity proteins for all the toxins (open circles). Rap2a dimers (purple) form a protective barrier in the periplasm, rapidly binding and sequestering Ssp2. C. When a Δ*rap2a* mutant of Db10 is the target strain, all of the effectors except for Ssp2 are still neutralized, but Ssp2 secreted by wild type cells is now able to cause toxicity in the target. D. In a putative ancestral cell, T6SS secretion of Ssp2, but not other toxins later acquired by horizontal transfer, inhibits a competitor target cell. In this case, loss of Ssp2 (or resistance to Ssp2 in the target) would prevent inhibition. For clarity, Ssp1 and Rap1a are not shown but would behave similarly to Ssp2 and Rap2a.

In summary, our data support a model (Fig. 8) whereby Ssp1 and Ssp2 are toxins secreted by the *S. marcescens* T6SS into the periplasm of a neighbouring cell. If this is an isogenic sibling, the cognate Rap proteins provide an efficient protective barrier, effectively binding and sequestering the toxin, whereas if it is a competitor, Ssp1/2 are free to attack the cell wall. However, Ssp1 and Ssp2 are only two of multiple distinct T6-secreted toxins. This provides the observed redundancy of function: if Ssp1/2 are missing, the other toxins still cause the efficient death of a competitor. This ‘belt and braces’ approach provides great robustness: if a competitor becomes resistant to one or two toxins, the others will still provide the ability to kill or inhibit the competitor efficiently. Of course, the toxic effect of Ssp1/2 can be seen against self (if the cognate Rap is missing) as Db10 has resistance proteins to neutralize the other toxins. We believe that other effectors most likely act on different cellular targets, analogous to the *Pseudomonas*-specific, cytoplasmic-acting toxin, Tse2 (Li *et al*., 2012). Finally, we speculate that as the Ssp and Rap proteins are encoded within the T6SS gene cluster, and related genes are found associated with other T6SSs, particularly closely related ones, they may represent the ancestral substrates of the *S. marcescens* T6SS. Subsequently, other toxins could have been horizontally acquired from diverse sources and adopted by this highly versatile secretion machine. The exciting task of deciphering how such species- and strain-specific arsenals of toxins and immunity proteins are co-ordinated by the producing cell and how they contribute to the dynamic composition of polymicrobial communities in infection and the environment lies ahead.

## Experimental procedures

### Bacterial strains, plasmids and culture conditions

All strains and plasmids used in this study are detailed in Table S1. All mutants constructed in *S. marcescens* Db10 were in-frame deletion mutants, generated by allelic exchange as described previously (Murdoch *et al*., 2011). Streptomycin-resistant derivatives were generated by phage ϕIF3-mediated transduction of the resistance allele from *S. marcescens* Db11 (Petty *et al*., 2006). Plasmids for constitutive expression of proteins in *S. marcescens* were derived from pSUPROM, plasmids for arabinose-inducible protein expression were derived from pBAD18-Kn, and derivatives of the pET15b-TEV plasmid were generated for protein overexpression and purification. *S. marcescens* was grown at 30°C in LB (10 g l^−1^ tryptone, 5 g l^−1^ yeast extract, 10 g l^−1^ NaCl, with 1.5 g l^−1^ agar for solid media) or minimal media (40 mM K_2_HPO_4_, 15 mM KH_2_PO_4_, 0.1% (NH_4_)_2_SO_4_, 0.4 mM MgSO_4_, 0.2% glucose) and *E. coli* was normally grown at 37°C in LB or M9 minimal media (M9). M9 (Sambrook and Russell, 2001) contained 0.5% glycerol (plus stated concentrations of arabinose and glucose as required). Growth analyses were performed in 96-well plates in a BioTek Synergy platereader. When required, media were supplemented with antibiotics: ampicillin (Ap) 100 μg ml^−1^, kanamycin (Kn) 100 μg ml^−1^, streptomycin (Sm) 100 μg ml^−1^, chloramphenicol (Cm) 25 μg ml^−1^; to maintain repression of proteins expressed from pBAD18-Kn, 0.5% glucose was added to the media for cloning and maintenance.

### Antibacterial competition/co-culture assays

These were based on the assay described previously (Murdoch *et al*., 2011). In brief, the attacker strain and target strain (both at OD_600_ 0.5) were mixed at an initial ratio of 1:1 attacker : target, co-cultured on solid LB for 7.5 h at 30°C and then the surviving target cells enumerated by serial dilution and viable counts on streptomycin-containing media. The target strain was always the streptomycin-resistant version of the mutant in question (Table S1). Statistical significance testing was performed by ANOVA followed by Dunnett's post test (GraphPad Prism software).

Bioinformatic identification of homologues of Rap and Ssp proteins and determination of their genetic contexts utilized the blast servers and sequence databases at the NCBI (http://www.ncbi.nlm.nih.gov).

### Immunodetection of secreted proteins

Anti-Hcp immunoblots were performed as described (Murdoch *et al*., 2011). For detection of Ssp1 and Ssp2, cellular and secreted fractions were prepared from 25 ml of culture grown for 7 h in LB. Secreted proteins were precipitated using 50:50 chloroform : methanol followed by methanol wash and resuspension in 2× gel sample buffer (Murdoch *et al*., 2011). Cellular samples were prepared by sonicating harvested cells in 20 mM Tris·HCl pH 7.5, 150 mM NaCl, 1 mM EDTA, 0.5% Triton X-100 and isolating the soluble fraction by centrifugation. Anti-Ssp1 and anti-Ssp2 rabbit polyclonal antibodies were raised to the purified proteins (Eurogentec, Belgium) and used at 1:1000; peroxidase-conjugated secondary (Thermo Fisher Scientific) was used at 1:10 000. Anti-RNAP β (Neoclone, USA) was used at 1:20 000, with anti-mouse secondary (Roche) at 1:10 000. In all cases, protein from the same number of cells was loaded in the secreted versus the cellular samples.

### Localization of Rap-HA proteins

Fractionation was performed using a cold osmotic shock procedure. Following growth of cultures for 5 h in LB, Tris·HCl pH 7.8 was added to 5 ml of cells to a final concentration of 50 mM and the cells were incubated for 10 min at room temperature, then recovered by centrifugation and washed once in LB. Cell pellets were resuspended in 1 ml of 40% sucrose, 30 mM Tris·HCl pH 7.8, 2 mM EDTA and incubated for 10 min at 30°C. One hundred microlitres of this fraction (‘whole cell’) was removed for analysis. Remaining cells were recovered by centrifugation, resuspended in 900 μl of ice-cold water and incubated on ice for 10 min, resulting in the release of the periplasm. After centrifugation, 100 μl of the supernatant (‘periplasm’ fraction) was retained for analysis. The pellet, containing the ‘cytoplasm + membranes’ fraction, was resuspended in 900 μl of 50 mM Tris·HCl pH 7.8 and 100 μl retained for analysis. Equivalent amounts, on a per cell basis, of each fraction in each strain were assayed. Anti-RNAP β was used as above, anti-MBP (NEB) was used at 1:10 000 and anti-HA (Roche) was used at 1:6000, all with anti-mouse secondary as above.

### Microscopic analysis of colony and cell morphology

Overnight cultures were normalized to OD_600_ 0.5, diluted 10^−2^ (culture spots) or to obtain single colonies, 10 μl spotted onto solid media and grown for 24 or 48 h. Macroscopic morphology of culture spots and single colonies were recorded using a Zeiss MZ16FA Stereo Microscope with a Leica DFC350 FX camera and Leica AF6000 software. Microscopic analysis of single cells taken from spots grown on solid media as above was performed by Differential Interference Contrast (DIC) microscopy using an Axioskop 2 mot plus (Zeiss) with a SPOT RT KE camera and spot software (Diagnostic Instruments).

### Protein purification and *in vitro* analysis

A full description of protein purification is provided in the *Supporting information*. In brief, proteins were overproduced in *E. coli* with a TEV protease-cleavable His_6_ tag and isolated by Ni^2+^ affinity purification. If required, the His_6_ tag was cleaved using TEV protease and the protein re-isolated by reverse Ni^2+^ chromatography. A final SEC step was then always performed. For SEC analysis of complex formation, His_6_-tagged proteins (10 nmol each) in 50 mM Tris·HCl, pH 7.5, 250 mM NaCl were separated on a Superdex 75 10/300 GL column, calibrated using molecular weight standards (GE Healthcare). Quantitative SYPRO Orange staining was performed as described (Rickman *et al*., 2004), with image analysis using ImageJ. Molar ratios were the mean of at least four quantifications. ITC was performed in 50 mM Tris·HCl, pH 7.5, 250 mM NaCl at 30°C in a MicroCal iTC200 calorimeter. The sample cell contained 6 μM Rap1a dimer or Rap2a dimer and the syringe contained 120 μM Ssp1 or Ssp2. Titrations consisted of 30 × 8 μl injections of Ssp into Rap (or into buffer alone as control). Data analysis was performed with the Origin software provided (MicroCal). For affinity isolation of Ssp1 and Ssp2, 10 μg of His_6_-tagged Rap protein was immobilized on magnetic Ni^2+^ beads (Qiagen), incubated for 1 h with culture supernatant (after 7 h growth in LB), beads washed and bound proteins eluted by the addition of gel sample buffer. Identified proteins were excised from the Coomassie- (Ssp2) or Silver- (Ssp1) stained gel and identified by mass spectrometry.

### Crystallographic analyses

Well-ordered trigonal and orthorhombic crystals of Rap1b and Rap2b, respectively, were obtained. The asymmetric unit for Rap1b consisted of a single subunit, while Rap2b displayed two dimers in the asymmetric unit. Diffraction data were recorded in-house and experimental phases were derived by single-wavelength anomalous diffraction measurements (Micossi *et al*., 2002), exploiting the signal from endogenous S atoms and I^−^ ions that had been added by soaking. The electron density maps were of high quality, and the models were completed, then refined to high resolution using standard methods (Dawson *et al*., 2008). Full crystallographic details are provided in the *Supporting information* and Table 1. Atomic co-ordinates and structure factors have been deposited in the Protein Data Bank (PDB) with accession codes 4AX2 (Rap1b) and 4B6I (Rap2b).

## References

[b1] Aubert DF, Flannagan RS, Valvano MA (2008). A novel sensor kinase-response regulator hybrid controls biofilm formation and type VI secretion system activity in *Burkholderia cenocepacia*. Infect Immun.

[b2] Basler M, Pilhofer M, Henderson GP, Jensen GJ, Mekalanos JJ (2012). Type VI secretion requires a dynamic contractile phage tail-like structure. Nature.

[b3] Bonemann G, Pietrosiuk A, Mogk A (2010). Tubules and donuts: a type VI secretion story. Mol Microbiol.

[b4] Burtnick MN, Brett PJ, Harding SV, Ngugi SA, Ribot WJ, Chantratita N (2011). The cluster 1 type VI secretion system is a major virulence determinant in *Burkholderia pseudomallei*. Infect Immun.

[b5] Cascales E (2008). The type VI secretion toolkit. EMBO Rep.

[b6] Cascales E, Cambillau C (2012). Structural biology of type VI secretion systems. Philos Trans R Soc Lond B Biol Sci.

[b7] Choi SH, Lee JE, Park SJ, Kim MN, Choo EJ, Kwak YG (2007). Prevalence, microbiology, and clinical characteristics of extended-spectrum beta-lactamase-producing *Enterobacter* spp., *Serratia marcescens**Citrobacter freundii*, and *Morganella morganii* in Korea. Eur J Clin Microbiol Infect Dis.

[b1001] Chou S, Bui NK, Russell AB, Lexa KW, Gardiner TE, Leroux M, Vollmer W, Mougous JD (2012). Structure of a peptidoglycan amidase effector targeted to Gram-negative bacteria by the Type VI secretion system. Cell Rep.

[b8] Cruickshank DW (1999). Remarks about protein structure precision. Acta Crystallogr D Biol Crystallogr.

[b9] Dawson A, Fyfe PK, Hunter WN (2008). Specificity and reactivity in menaquinone biosynthesis: the structure of *Escherichia coli* MenD (2-succinyl-5-enolpyruvyl-6-hydroxy-3-cyclohexadiene-1-carboxylate synthase). J Mol Biol.

[b11] Ding J, Wang W, Feng H, Zhang Y, Wang DC (2012). Structural insights into the *Pseudomonas aeruginosa* type VI virulence effector Tse1 bacteriolysis and self-protection mechanisms. J Biol Chem.

[b12] Filloux A, Hachani A, Bleves S (2008). The bacterial type VI secretion machine: yet another player for protein transport across membranes. Microbiology.

[b13] Gerlach RG, Hensel M (2007). Protein secretion systems and adhesins: the molecular armory of Gram-negative pathogens. Int J Med Microbiol.

[b14] Hood RD, Singh P, Hsu F, Guvener T, Carl MA, Trinidad RR (2010). A type VI secretion system of *Pseudomonas aeruginosa* targets a toxin to bacteria. Cell Host Microbe.

[b15] Jani AJ, Cotter PA (2010). Type VI secretion: not just for pathogenesis anymore. Cell Host Microbe.

[b16] Kelley LA, Sternberg MJ (2009). Protein structure prediction on the Web: a case study using the Phyre server. Nat Protoc.

[b17] Krissinel E, Henrick K (2007). Inference of macromolecular assemblies from crystalline state. J Mol Biol.

[b18] Laskowski RA, Watson JD, Thornton JM (2005). ProFunc: a server for predicting protein function from 3D structure. Nucleic Acids Res.

[b19] Leiman PG, Basler M, Ramagopal UA, Bonanno JB, Sauder JM, Pukatzki S (2009). Type VI secretion apparatus and phage tail-associated protein complexes share a common evolutionary origin. Proc Natl Acad Sci USA.

[b20] Li M, Le Trong I, Carl MA, Larson ET, Chou S, De Leon JA (2012). Structural basis for type VI secretion effector recognition by a cognate immunity protein. PLoS Pathog.

[b21] Lockhart SR, Abramson MA, Beekmann SE, Gallagher G, Riedel S, Diekema DJ (2007). Antimicrobial resistance among Gram-negative bacilli causing infections in intensive care unit patients in the United States between 1993 and 2004. J Clin Microbiol.

[b22] MacIntyre DL, Miyata ST, Kitaoka M, Pukatzki S (2010). The *Vibrio cholerae* type VI secretion system displays antimicrobial properties. Proc Natl Acad Sci USA.

[b23] Micossi E, Hunter WN, Leonard GA (2002). De novo phasing of two crystal forms of tryparedoxin II using the anomalous scattering from S atoms: a combination of small signal and medium resolution reveals this to be a general tool for solving protein crystal structures. Acta Crystallogr D Biol Crystallogr.

[b24] Mulder DT, Cooper CA, Coombes BK (2012). Type VI secretion system-associated gene clusters contribute to pathogenesis of *Salmonella enterica* serovar Typhimurium. Infect Immun.

[b25] Murdoch SL, Trunk K, English G, Fritsch MJ, Pourkarimi E, Coulthurst SJ (2011). The opportunistic pathogen *Serratia marcescens* utilizes type VI secretion to target bacterial competitors. J Bacteriol.

[b10] de Pace F, Nakazato G, Pacheco A, de Paiva JB, Sperandio V, da Silveira WD (2010). The type VI secretion system plays a role in type 1 fimbria expression and pathogenesis of an avian pathogenic *Escherichia coli* strain. Infect Immun.

[b26] Petersen TN, Brunak S, von Heijne G, Nielsen H (2011). SignalP 4.0: discriminating signal peptides from transmembrane regions. Nat Methods.

[b27] Petty NK, Foulds IJ, Pradel E, Ewbank JJ, Salmond GP (2006). A generalized transducing phage (phiIF3) for the genomically sequenced *Serratia marcescens* strain Db11: a tool for functional genomics of an opportunistic human pathogen. Microbiology.

[b28] Pukatzki S, Ma AT, Revel AT, Sturtevant D, Mekalanos JJ (2007). Type VI secretion system translocates a phage tail spike-like protein into target cells where it cross-links actin. Proc Natl Acad Sci USA.

[b29] Pukatzki S, McAuley SB, Miyata ST (2009). The type VI secretion system: translocation of effectors and effector-domains. Curr Opin Microbiol.

[b30] Rickman C, Meunier FA, Binz T, Davletov B (2004). High affinity interaction of syntaxin and SNAP-25 on the plasma membrane is abolished by botulinum toxin E. J Biol Chem.

[b31] Rosales-Reyes R, Skeldon AM, Aubert DF, Valvano MA (2012). The Type VI secretion system of *Burkholderia cenocepacia* affects multiple Rho family GTPases disrupting the actin cytoskeleton and the assembly of NADPH oxidase complex in macrophages. Cell Microbiol.

[b32] Russell AB, Hood RD, Bui NK, LeRoux M, Vollmer W, Mougous JD (2011). Type VI secretion delivers bacteriolytic effectors to target cells. Nature.

[b33] Russell AB, Singh P, Brittnacher M, Bui NK, Hood RD, Carl MA (2012). A widespread bacterial type VI secretion effector superfamily identified using a heuristic approach. Cell Host Microbe.

[b34] Sambrook J, Russell DW (2001). Molecular Cloning: A Laboratory Manual.

[b35] Schwarz S, West TE, Boyer F, Chiang WC, Carl MA, Hood RD (2010). *Burkholderia* type VI secretion systems have distinct roles in eukaryotic and bacterial cell interactions. PLoS Pathog.

[b36] Suarez G, Sierra JC, Erova TE, Sha J, Horneman AJ, Chopra AK (2010). A type VI secretion system effector protein, VgrG1, from *Aeromonas hydrophila* that induces host cell toxicity by ADP ribosylation of actin. J Bacteriol.

[b37] Wiseman T, Williston S, Brandts JF, Lin LN (1989). Rapid measurement of binding constants and heats of binding using a new titration calorimeter. Anal Biochem.

[b38] Yamaguchi Y, Inouye M (2011). Regulation of growth and death in *Escherichia coli* by toxin-antitoxin systems. Nat Rev Microbiol.

[b39] Zheng J, Leung KY (2007). Dissection of a type VI secretion system in *Edwardsiella tarda*. Mol Microbiol.

[b40] Zou T, Yao X, Qin B, Zhang M, Cai L, Shang W (2012). Crystal structure of *Pseudomonas aeruginosa* Tsi2 reveals a stably folded superhelical antitoxin. J Mol Biol.

