# Supplementary material for: New secreted toxins and immunity proteins encoded within the Type VI secretion system gene cluster of Serratia marcescens
Source: Mol Microbiol. 2012 Sep 27;86(4):921–36. doi: 10.1111/mmi.12028 (PMC3533786; doi:10.1111/mmi.12028)
Supplement: Supplementary file 1 [file mmi0086-0921-SD1.pdf]

Supporting Information

New Secreted Toxins and Immunity Proteins Encoded within the Type VI Secretion System Gene Cluster of *Serratia marcescens*

Grant English[1,#], Katharina Trunk[1,#], Vincenzo A. Rao[2], Velupillai Srikannathasan[2], William N. Hunter[2] and Sarah J. Coulthurst[1,*]

[1]Division of Molecular Microbiology, [2]Division of Biological Chemistry and Drug Discovery, College of Life Sciences, University of Dundee, Dundee, UK.

[#]These authors contributed equally and are listed alphabetically

*For correspondence (s.j.coulthurst@dundee.ac.uk; tel +44 1382 386208; fax +44 1382 388216)

Figure S1

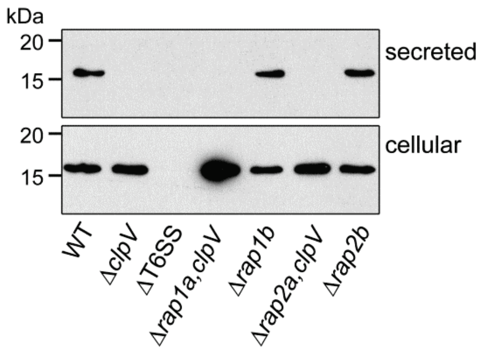

Fig. S1. Hcp secretion by *rap*, *ssp* and T6SS mutants.
Anti-Hcp1 immunoblot of secreted and cellular proteins produced by wild type *S. marcescens* Db10 (WT) and mutant strains: Δ*clpV*; ΔT6SS (Δ*SMA2244-2281*); Δ*rap1a,clpV*; Δ*rap1b*; Δ*rap2a,clpV*; and Δ*rap2b*. The amount of sample loaded for each fraction corresponded to the same number of cells.

Figure S2

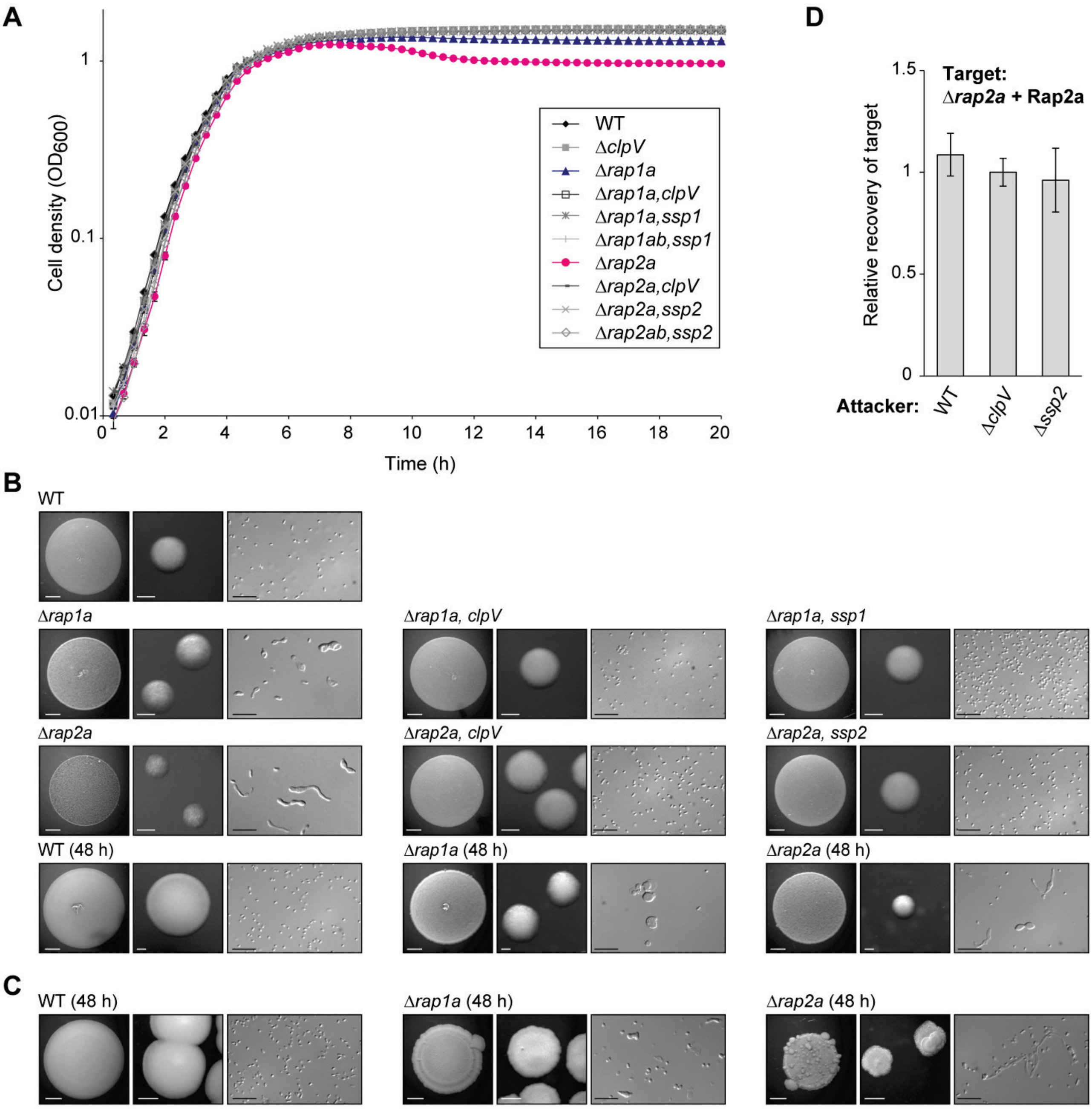

Fig. S2. Phenotypes of mutants lacking resistance proteins Rap1a and Rap2a.

A. Growth in liquid LB medium of wild type (WT) *S. marcescens* Db10 and mutants lacking the genes indicated. Points show mean of three replicates; error bars (SEM) are included for Δ*rap1a* and Δ*rap2a* but are mostly smaller than the points.

B. & C. Plate phenotypes of WT and selected mutants when (B) grown on solid minimal medium, for 24 h unless indicated otherwise; or (C) when grown on solid LB medium for 48 h. For each mutant, representative images of the morphology of a culture spot (left), single colonies (middle) and individual cells (right) are shown. Scale bars for minimal media (B) are 2 mm (culture spots) 200 μm (colonies) and 10 μm (cells). Scale bars for LB media (C) are 2 mm (culture spots), 1 mm (colonies) and 10 μm (cells).

D. Recovery of a Δ*rap2a* mutant (Δ*rap2a*,Δ*clpV*) carrying the complementing plasmid pSC542, following co-culture with the different attacking strains indicated, expressed relative to recovery of the target when co-cultured with the Δ*clpV* mutant. Points show mean +/- SEM (n = 4).

Figure S3.

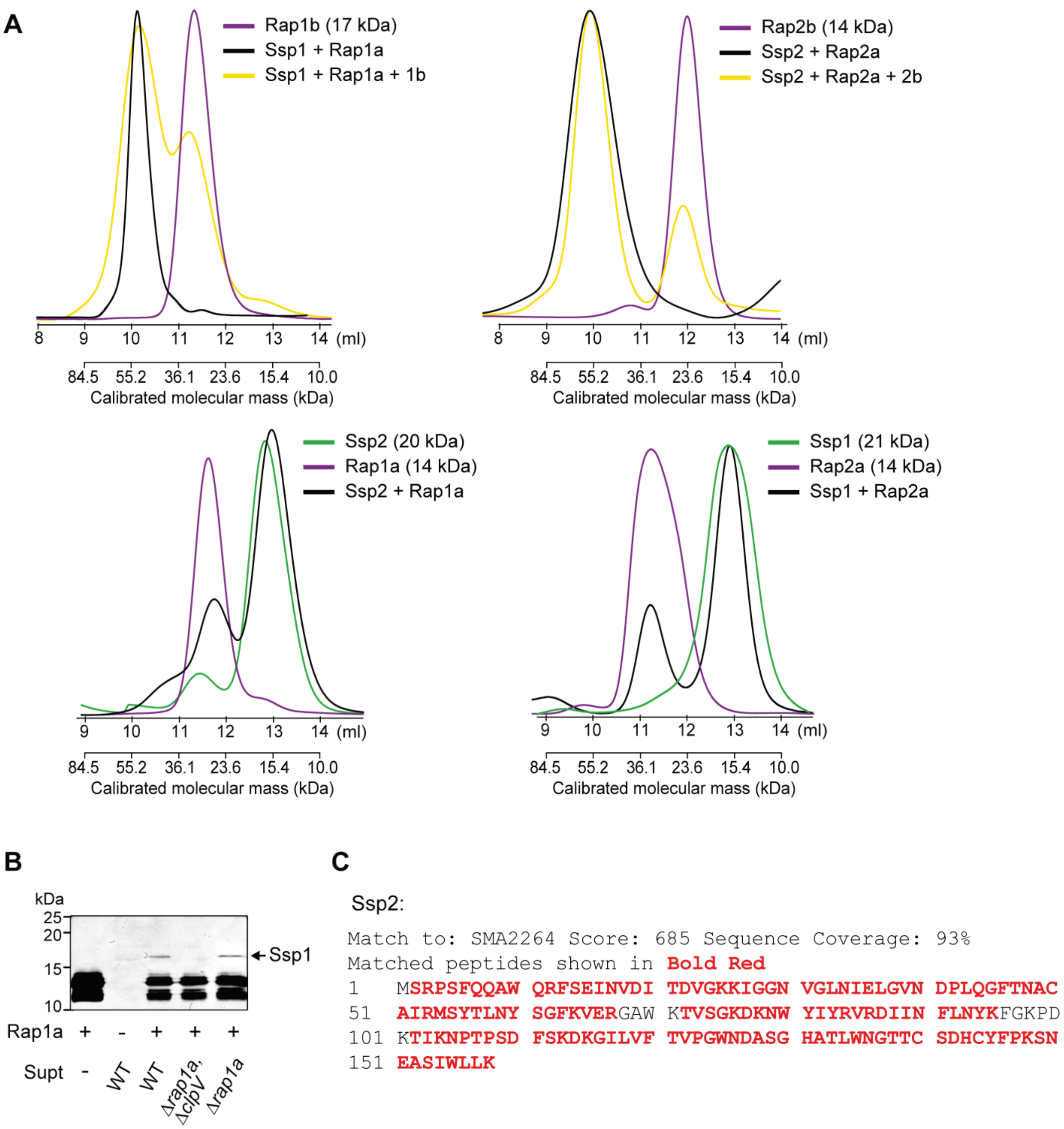

Fig S3. Interactions between Ssp and Rap proteins.

A. Size exclusion chromatography analyses of the interactions between Ssp1, Rap1a and Rap1b (top left), between Ssp2, Rap2a and Rap2b (top right), between Ssp2 and Rap1a (bottom left) and between Ssp1 and Rap2a (bottom right). In each case, 10 nmol of protein (or 10 nmol of each protein in the case of mixtures) was separated on a calibrated Superdex 75 10/300 GL column. The theoretical molecular mass of each monomer is given.

B. Affinity purification of secreted Ssp1 from culture supernatant (supt) derived from the strain indicated (WT, wild type *S. marcescens* Db10), using immobilised His-Rap1a as bait.

C. Sequence coverage obtained on mass spectrometric analysis of secreted Ssp2 isolated by affinity purification from culture supernatant using Rap2a as bait.

Figure S4.

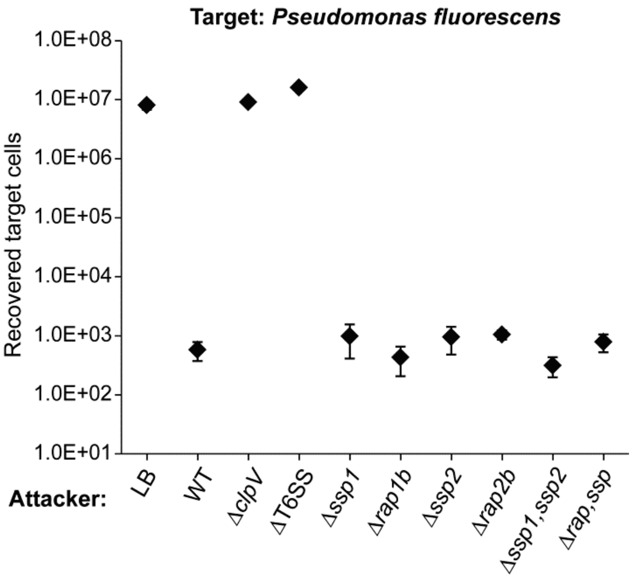

Fig. S4. T6SS-mediated anti-bacterial killing activity of *S. marcescens* against the target strain *Pseudomonas fluorescens*.

Recovery of viable *P. fluorescens* cells after 4 h co-culture with *S. marcescens*, at 30°C with an initial ratio of 5 *S. marcescens* : 1 *P. fluorescens*. The strains compared are *S. marcescens* Db10 (WT) and the mutants: Δ*clpV*; ΔT6SS (Δ*SMA2244-2281*); Δ*ssp1*; Δ*rap1b*; Δ*ssp2*; Δ*rap2b*; Δ*ssp1, ssp2;* and Δ*rap, ssp* (Δ*ssp1, rap1a, rap1b, ssp2, rap2a, rap2b*). LB, target with sterile media only. Points show mean +/- SEM (n ≥ 3).

Figure S5.

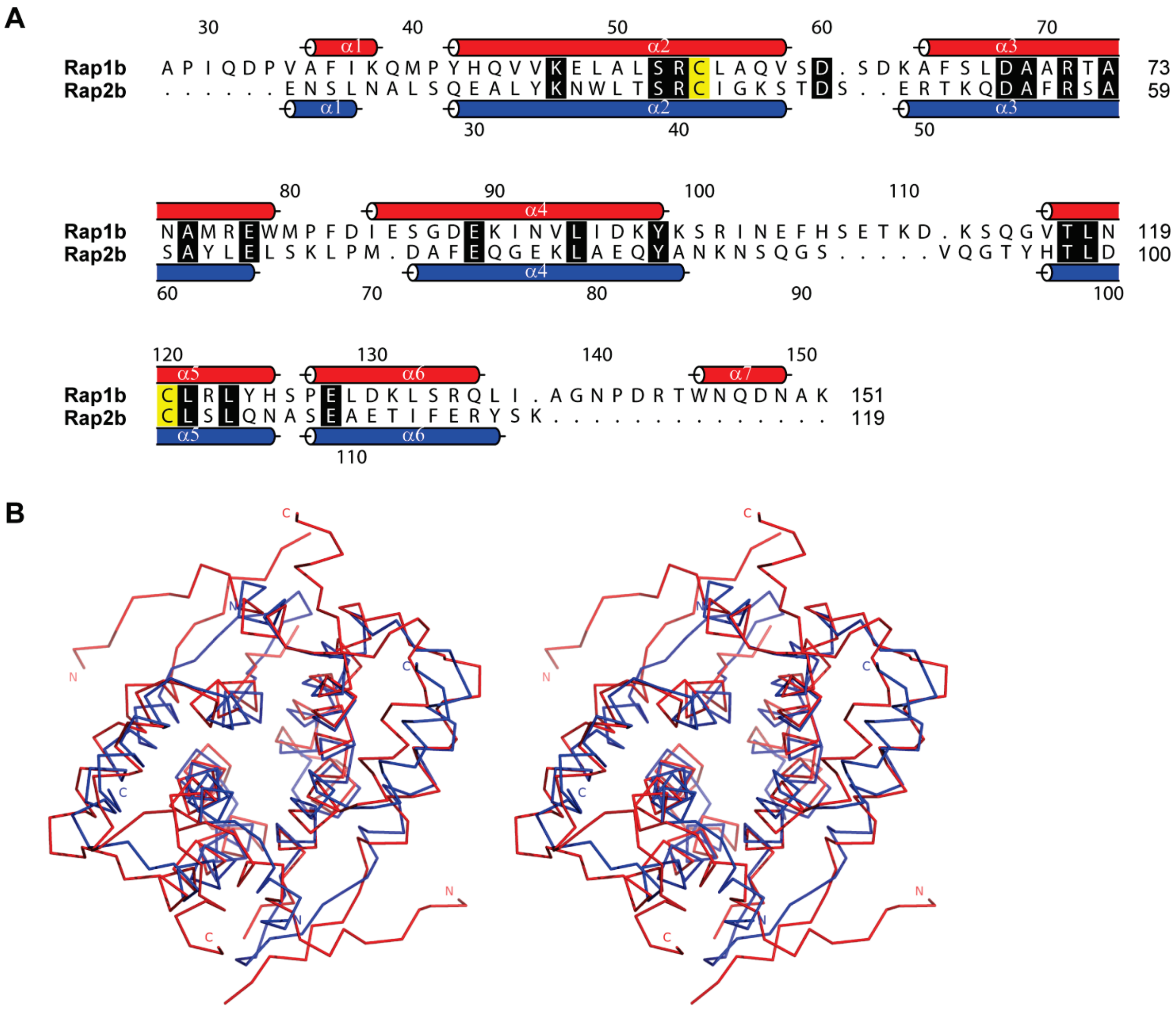

Fig. S5. Structural conservation of Rap1b and Rap2b.

A. Structure-based sequence alignment of the mature proteins highlights the conserved secondary structure content in Rap1b (red cylinders) and Rap2b (blue cylinders) despite the low sequence identity of 20 % between the two proteins. Residues involved in disulfide bond formation are coloured yellow.

B. Stereoview overlay of the Cα trace of Rap1b and Rap2b. Rap1b is coloured red and superimposed on Rap2b which is blue.

Table S1. Bacterial Strains and Plasmids used in this study.

Name	Description	Source or Reference
**Bacterial Strains**		
S. marcescens Db10	Wild type strain, non-pigmented	(Flyg *et al.*, 1980)
S. marcescens Db11	Sm-resistant derivative of Db10	(Kurz *et al.*, 2003)
SJC3	Db10 Δ*clpV* (ΔSMA2274)	(Murdoch *et al.*, 2011)
SJC11	Db10 Δ*tssE* (ΔSMA2271)	(Murdoch *et al.*, 2011)
SJC19	Db10 Δ*pppA* (ΔSMA2268)	This study
SJC30	Db10 Δ*ssp1* (ΔSMA2261)	This study
SJC31	Db10 Δ*rap1b* (ΔSMA2262)	This study
SJC32	Db10 Δ*ssp2* (ΔSMA2264)	This study
SJC33	Db10 Δ*rap1a* (ΔSMA2260)	This study
SJC35	Db10 Δ*rap2b* (ΔSMA2266)	This study
SJC36	Db10 Δ*rap2a,clpV* (ΔSMA2265,ΔSMA2274)	This study
SJC37	Db10 Δ*ssp1,ssp2* (ΔSMA2261,ΔSMA2264)	This study
SJC39	Db10 Δ*rap1a,clpV* (ΔSMA2260,ΔSMA2274)	This study
SJC40	Db10 Δ*rap2a* (ΔSMA2265)	This study
SJC42	Db10 Δ*rap1a,ssp1* (ΔSMA2260,ΔSMA2261)	This study
SJC43	Db10 Δ*rap2a,ssp2* (ΔSMA2265,ΔSMA2264)	This study
GE01	Db10 Δ*rap1ab,ssp1* (ΔSMA2260-2262)	This study
GE03	Db10 Δ*rap,ssp* (ΔSMA2260-2262, ΔSMA2264-2266)	This study
KT05	Sm-resistant derivative of wild type Db10	This study
KT06	Sm-resistant derivative of SJC3 (Δ*clpV*)	This study
KT08	Sm-resistant derivative of GE01 (Δ*rap1ab,ssp1*)	This study
KT10	Sm-resistant derivative of GE03 (Δ*rap,ssp*)	This study
KT13	Db10 ΔT6SS (ΔSMA2244-SMA2281)	This study
KT14	Db10 Δ*rap2ab,ssp2* (ΔSMA2264-2266)	This study
KT24	Sm-resistant derivative of KT13 (ΔT6SS)	This study
KT25	Sm-resistant derivative of KT14 (Δ*rap2ab,ssp2*)	This study
KT47	Sm-resistant derivative of SJC30 (Δ*ssp1*)	This study
KT48	Sm-resistant derivative of SJC31 (Δ*rap1b*)	This study
KT52	Sm-resistant derivative of SJC35 (Δ*rap2b*)	This study
KT53	Sm-resistant derivative of SJC36 (Δ*rap2a,clpV*)	This study
KT55	Sm-resistant derivative of SJC32 (Δ*ssp2*)	This study
KT60	Sm-resistant derivative of SJC39 (Δ*rap1a,clpV*)	This study
E. coli MG1655	Wild type (model K12 strain)	(Blattner *et al.*, 1997)
E. coli BL21(DE3) pLysS	Protein overexpression strain. Chromosomal λDE3 encodes IPTG-inducible T7 RNA polymerase. pLysS directs expression of T7 lysozyme.	Novagen
E. coli Rosetta-gami (DE3)	Protein overexpression strain. Chromosomal λDE3 encodes IPTG-inducible T7 RNA polymerase. Mutations in *gor*, *trxB*, *ahpC*.	Novagen
E. coli DH5*a*	Cloning host	Invitrogen
E. coli CC118λ*pir*	Cloning host and donor strain for pKNG101-derived marker exchange plasmids (λ*pir*)	(Herrero *et al.*, 1990)
E. coli HH26 pNJ5000	Mobilising strain for conjugal transfer	(Grinter, 1983)
KT02	Sm^R derivative of *P. fluorescens* 55	(Murdoch et al., 2011)
**Plasmids**		
pSUPROM	Vector for constitutive expression of cloned genes under the control of the *E. coli tat* promoter (Kn^R)	(Jack *et al.*, 2004)
pBAD18-Kn	Arabinose-inducible expression vector (Kn^R). Gene of interest cloned downstream of the P_{ara} promoter, with its own ribosome binding site	(Guzman *et al.*, 1995)
pET15b-TEV	Protein overexpression vector. Protein expressed under the control of the T7 promoter. Permits fusion of an His_6 tag followed by a TEV protease cleavage site to the N-terminus of the overexpressed protein,	(Rao *et al.*, 2011)

pBluescript KS+	High copy cloning vector (Ap^R)	Stratagene
pKNG101	Suicide vector for marker exchange (Sm^R, *sacBR*, mobRK2, ori R6K)	(Kaniga *et al.*, 1991)
pSC039	Coding sequence for ClpV (SMA2274) in pSUPROM	(Murdoch *et al.*, 2011)
pSC045	Coding sequence for TssE (SMA2271) in pSUPROM	(Murdoch *et al.*, 2011)
pSC133	Coding sequence for Ssp2 (SMA2264) in pBAD18-Kn	This study
pSC134	Coding sequences for Ssp2+ Rap2a + Rap2b (SMA2264-2266) in pBAD18-Kn	This study
pSC138	Coding sequence for an $OmpA_{SP}$-Ssp2 ($OmpA_{SP}$-SMA2264) fusion protein in pBAD18-Kn. $OmpA_{SP}$ is the *E. coli* OmpA signal peptide (aa 1-24).	This study
pSC144	Coding sequences for $OmpA_{SP}$-Ssp2 + Rap2a + Rap 2b ($OmpA_{SP}$-SMA2264 + SMA2265 + SMA2266) in pBAD18-Kn	This study
pSC151	Coding sequence for Ssp1 (SMA2261) in pBAD18-Kn	This study
pSC152	Coding sequence for an $OmpA_{SP}$-Ssp1 ($OmpA_{SP}$-SMA2261) fusion protein in pBAD18-Kn. $OmpA_{SP}$ is the *E. coli* OmpA signal peptide (aa 1-24).	This study
pSC159	Coding sequences for Rap1a + Ssp1+ Rap1b (SMA2260-2262) in pBAD18-Kn	This study
pSC160	Coding sequences for Rap1a + $OmpA_{SP}$-Ssp1 + Rap 1b (SMA2260 + $OmpA_{SP}$-SMA2261 + SMA2262) in pBAD18-Kn	This study
pSC501	Coding sequence for Rap1a minus signal peptide (SMA2260 amino acids 27-127) in pET15b-TEV, generating a fusion protein with a TEV-cleavable N-terminal His_6 tag.	This study
pSC502	Coding sequence for Ssp1 (SMA2261) in pET15b-TEV, generating a fusion protein with a TEV-cleavable N-terminal His_6 tag.	This study
pSC503	Coding sequence for Rap1b minus signal peptide (SMA2262 amino acids 28-151) in pET15b-TEV, generating a fusion protein with a TEV-cleavable N-terminal His_6 tag.	This study
pSC504	Coding sequence for Ssp2 (SMA2264) in pET15b-TEV, generating a fusion protein with a TEV-cleavable N-terminal His_6 tag.	This study
pSC505	Coding sequence for Rap2a minus signal peptide (SMA2265 amino acids 25-124) in pET15b-TEV, generating a fusion protein with a TEV-cleavable N-terminal His_6 tag.	This study
pSC506	Coding sequence for Rap2b minus signal peptide (SMA2266 amino acids 21-119) in pET15b-TEV, generating a fusion protein with a TEV-cleavable N-terminal His_6 tag.	This study
pSC538	Coding sequence for Rap1a (including signal peptide, SMA2260) with a C-terminal HA tag in pSUPROM	This study
pSC541	Coding sequence for Ssp2 (SMA2264) with a C-terminal HA tag in pSUPROM	This study
pSC542	Coding sequence for Rap2a (including signal peptide, SMA2265) with a C-terminal HA tag in pSUPROM	This study
pSC543	Coding sequence for Rap2b (including signal peptide, SMA2266) with a C-terminal HA tag in pSUPROM	This study
pSC105	pKNG101-derived marker exchange plasmid for the generation of chromosomal Δ*SMA2268* (Δ*pppA*)	This study
pSC122	pKNG101-derived marker exchange plasmid for the generation of chromosomal Δ*SMA2260* (Δ*rap1a*)	This study
pSC123	pKNG101-derived marker exchange plasmid for the generation of chromosomal Δ*SMA2261* (Δ*ssp1*)	This study
pSC124	pKNG101-derived marker exchange plasmid for the generation of chromosomal Δ*SMA2262* (Δ*rap1b*)	This study
pSC125	pKNG101-derived marker exchange plasmid for the generation of chromosomal Δ*SMA2264* (Δ*ssp2*)	This study
pSC126	pKNG101-derived marker exchange plasmid for the generation of chromosomal Δ*SMA2265* (Δ*rap2a*)	This study
pSC127	pKNG101-derived marker exchange plasmid for the generation of chromosomal Δ*SMA2266* (Δ*rap2b*)	This study

pSC145	pKNG101-derived marker exchange plasmid for the generation of chromosomal Δ*SMA2260-2261*(Δ*rap1a,ssp1*)	This study
pSC148	pKNG101-derived marker exchange plasmid for the generation of chromosomal Δ*SMA2264-2265*(Δ*ssp1,rap2a*)	This study
pSC519	pKNG101-derived marker exchange plasmid for the generation of chromosomal Δ*SMA2260-2262,2264-2266* (Δ*rap,ssp*). Contains three adjacent sequences: region upstream of *SMA2260*, *SMA2263*, region downstream of *SMA2266*; allowing one or both sets of three genes to be removed in-frame, depending on the recombination event but always leaving *SMA2263* intact.	This study
pSC1208	pKNG101-derived marker exchange plasmid for the generation of chromosomal Δ*SMA2244-2281* (ΔT6SS)	This study
pSC1209	pKNG101-derived marker exchange plasmid for the generation of chromosomal Δ*SMA2264-2266* (Δ*rap2ab,ssp2*)	This study

Supporting Experimental Procedures: Detailed protocols for protein purification and crystallography

Protein overproduction and purification

Recombinant Rap proteins were produced in *E. coli* Rosetta-gami (DE3) transformed with plasmids pSC501, pSC503, pSC505, or pSC506, and recombinant Ssp proteins were produced in *E. coli* BL21(DE3) pLysS transformed with plasmids pSC502 or pSC504. Overnight cultures of 50 ml LB media supplemented with Ap (Rap proteins) or Ap + Cm (Ssp proteins) were grown overnight at 37 °C and used to inoculate 950 ml LB media with Ap or Ap + Cm. Cultures were then grown for 2 h at 37 °C before inducing with isopropyl β-D-1-thiogalactopyranoside to a final concentration of 1 mM and growing at 19 °C for a further 24 h (Rap) or 18 h (Ssp). Cells were harvested by centrifugation (4,000 *g* at 4 °C for 40 min). The cell pellet was resuspended in 25 mM Tris-HCl pH 7.5, (1 mM DTT for the Rap proteins), supplemented with an EDTA-free protease inhibitor cocktail tablet (Calbiochem). Cells were lysed by passage through a French press at 1000 psi and cell debris removed following centrifugation (35,000 *g* at 4 °C for 30 min). His_6-tagged Rap and Ssp proteins were isolated by affinity chromatography using a 5 ml HisTrap HP column (GE Healthcare) pre-charged with Ni^{2+} and equilibrated in buffer A (25 or 50 mM Tris-HCl pH 7.5, 0.25 M NaCl, (1 mM DTT for Rap proteins), 25 mM imidazole). A linear concentration gradient of imidazole was applied to elute the protein using buffer B (25 or 50 mM Tris-HCl, pH 7.5, 0.25 M NaCl, (1 mM DTT for Rap proteins), 0.5 M imidazole). If required, Rap proteins were then dialysed against buffer C (25 mM Tris-HCl pH 7.5, 0.25 M NaCl, 1 mM DTT) at 4 °C. Fractions were analysed by sodium dodecyl sulfate polyacrylamide gel electrophoresis (SDS-PAGE) and those containing the wanted protein were pooled. For optional cleavage of the N-terminal His_6-tag, the protein was incubated overnight at 4 °C with His-tagged tobacco etch virus (TEV) protease and then loaded onto a HisTrap HP column and eluted as described above; untagged wanted protein did not bind to the column (reverse purification). All proteins were then further purified by size exclusion chromatography (SEC) using either a Superdex 75 26/60 column or a Superdex 75 10/300 GL column (GE Healthcare) and buffer comprising 50 mM Tris-HCl, 0.25 mM NaCl, (1 mM DTT for Rap). The columns had previously been calibrated with high and low molecular weight standards: blue dextran (> 2,000 kDa), thyroglobulin (669 kDa), ferritin (440 kDa), aldolase (158 kDa), conalbumin (75 kDa), ovalbumin (43 kDa), carbonic anhydrase (29.5 kDa), ribonuclease A (13.7 kDa) and aprotinin (6.5 kDa) (GE Healthcare). Fractions containing the wanted protein were pooled and concentrated using Amicon Ultra devices

(Millipore). Protein purity was confirmed by SDS-PAGE and mass spectrometry (Fingerprint Proteomics Facility, University of Dundee) and theoretical extinction coefficients (*PROTPARAM*, Gasteiger *et al.*, 2005) used to estimate protein concentration.

Diffracting crystals were obtained for His_6-tagged Rap1b. However, the affinity tag had to be removed to grow ordered crystals of Rap2b. All biochemical studies reported on Rap and Ssp proteins were performed in 50 mM Tris-HCl pH 7.5, 0.25 M NaCl using the His_6-tagged proteins. The oligomeric state was unaffected by the presence or otherwise of the His_6-tag.

Crystallization, data collection and structure determination

i) Rap1b - Crystallization screens of Rap1b were carried out at 20°C by the sitting drop vapor diffusion method in 96-well plates. This was achieved with a Phoenix Liquid Handling System (Rigaku, Art Robins Instruments) and the commercially available screens PEG (Qiagen) and JCSG+ (Molecular Dimensions). A promising condition was optimized using hanging drops consisting of 1 μl of protein at 8 mg/ml in 25 mM Tris-HCl, pH 7.5, 250 mM NaCl, 1 mM DTT and 1 μl of the reservoir 0.55 M Li_2SO_4, 5 % PEG 8000. Prismatic crystals attained maximum dimensions of approximately 0.25 mm, over five days. A crystal was soaked for approximately 20 seconds in 125 mM aqueous sodium iodide, and flash cooled to -190°C in a stream of nitrogen. No additional cryo-protectant was required. A dataset was measured using a Rigaku MicroMax 007 rotating-anode X-ray generator (copper K_α, λ=1.5418 Å) coupled to an R-AXIS IV^{++} image plate detector. All data were indexed and integrated using *MOSFLM* (Leslie, 2006), scaled and the space group checked using *SCALA* and *POINTLESS* (Evans, 2006) from the *CCP4* program suite (Winn *et al.*, 2011). The crystals display space group $P3_121$ with unit cell lengths a=b= 77.9 Å and c= 50.6 Å. The asymmetric unit consists of a single polypeptide chain with an estimated solvent content of 55 % and a V_m of 2.72 $Å^3$/Da.

This dataset was used to solve the structure by single-wavelength anomalous dispersion (SAD) methods targeting sulfur present in the amino acid sequence (2 cysteines and 4 methionines) and any iodides that were introduced. The positions of 5 S atoms and 2 I^- ions were identified using *PHENIX* (Adams *et al.*, 2010)and experimental phases were calculated in *PHASER* (McCoy *et al.*, 2007). The figure-of-merit was 0.28 for data to 1.9 Å resolution. The initiating methionine of the protein construct was subsequently modelled as two rotamers and such disorder is the likely reason for not finding this particular S atom using the anomalous differences. Density modification was carried out using solvent flattening and histogram matching, and initial model building was carried out using *RESOLVE* (Terwilliger, 2003). Refinement calculations were performed using *REFMAC5* (Murshudov *et al.*, 2011) and translation/libration/screw analysis (TLS) was applied (Winn *et al.*, 2001). Inspection of the model and the fit to electron and difference density maps was carried out in *COOT* (Emsley & Cowtan, 2004). *MolProbity* (Chen *et al.*, 2010) was used to investigate model geometry in combination with the validation tools provided in *COOT*. The analysis of secondary structure and surface interactions were performed using *DSSP* (Kabsch & Sander, 1983) and *PISA* (Krissinel & Henrick, 2007) respectively. Electrostatic surface representations were calculated using the *APBS* plugin for PyMOL (Baker *et al.*, 2001, DeLano, 2002).

ii) Rap2b - Crystallization screening of Rap2b was carried out as described for Rap1b, with

crystallization occurring at a protein concentration of 7.5 mg/ml and using the optimized reservoir condition 1.0 M LiCl, 20 % PEG 6000, 0.1 M citric acid pH 4.0. Block-like crystals with maximum dimensions of approximately 0.2 mm, grew over four days. A cryo-protectant was made by adjusting the mother liquor to incorporate 20% glycerol and the crystal was passed through this liquid, cooled and then used to measure a 2.4Å resolution data set (data set I). Another crystal was subsequently obtained and provided data to higher-resolution (2.0 Å data set II). The crystals of Rap2b display space group $P222_1$ with unit cell lengths a= 48.1 Å b= 57.0 Å and c= 122.4 Å. The asymmetric unit consists of four polypeptide chains, labelled A – D, with an estimated solvent content of 35 % and a V_m of 1.86 $Å^3$/Da.

Data set I was used to solve the structure by SAD methods again targeting endogenous S (8 cysteine and 8 methionine residues in the asymmetric unit) and any ordered halide ions. Six well-ordered S atoms were identified giving an initial figure-of-merit of 0.43 to 2.4Å resolution. A model of 353 residues with R_{work} and R_{free} values of 27.8 % and 33.8 % respectively was obtained. At this point another data set II became available and since this increased the resolution to 2.0 Å it was used to continue the analysis. Data were collected, processed, analyzed and the model refined as described above for Rap1b, with the inclusion of strict non-crystallographic symmetry (NCS) restraints in the early stages of the refinement. Crystallographic statistics are summarized in Table S5.

Molecular images were prepared using *PYMOL* (DeLano, 2002). Sequence alignments were determined using *MUSCLE* (Edgar, 2004) or T-Coffee (Di Tommaso *et al.*, 2011) and presented with *ALINE* (Bond & Schuttelkopf, 2009). Structural overlays were calculated using *SSM* (Krissinel & Henrick, 2004). Secondary structure predictions *de novo* were made using *PSIPRED* (Buchan *et al.*, 2010).

References

Adams, P. D., P. V. Afonine, G. Bunkóczi, V. B. Chen, I. W. Davis, N. Echols, J. J. Headd, L.-W. Hung, G. J. Kapral, R. W. Grosse-Kunstleve, A. J. McCoy, N. W. Moriarty, R. Oeffner, R. J. Read, D. C. Richardson, J. S. Richardson, T. C. Terwilliger & P. H. Zwart, (2010) PHENIX: a comprehensive Python-based system for macromolecular structure solution. *Acta Crystallogr D Biol Crystallogr* **66**: 213-221.

Baker, N. A., D. Sept, S. Joseph, M. J. Holst & J. A. McCammon, (2001) Electrostatics of nanosystems: application to microtubules and the ribosome. *Proceedings of the National Academy of Sciences of the United States of America* **98**: 10037-10041.

Blattner, F. R., G. Plunkett, 3rd, C. A. Bloch, N. T. Perna, V. Burland, M. Riley, J. Collado-Vides, J. D. Glasner, C. K. Rode, G. F. Mayhew, J. Gregor, N. W. Davis, H. A. Kirkpatrick, M. A. Goeden, D. J. Rose, B. Mau & Y. Shao, (1997) The complete genome sequence of *Escherichia coli* K-12. *Science* **277**: 1453-1462.

Bond, C. S. & A. W. Schuttelkopf, (2009) ALINE: a WYSIWYG protein-sequence alignment editor for publication-quality alignments. *Acta Crystallogr D Biol Crystallogr* **65**: 510-512.

Buchan, D. W., S. M. Ward, A. E. Lobley, T. C. Nugent, K. Bryson & D. T. Jones, (2010) Protein annotation and modelling servers at University College London. *Nucleic Acids Res* **38**: W563-568.

Chen, V. B., W. B. Arendall, J. J. Headd, D. A. Keedy, R. M. Immormino, G. J. Kapral, L. W. Murray, J. S. Richardson & D. C. Richardson, (2010) MolProbity: all-atom structure validation for macromolecular crystallography. *Acta Crystallogr D Biol Crystallogr* **66**: 12-21.

DeLano, W. L., (2002) The PyMOL Molecular Graphics System. In: http://www.pymol.org.

Di Tommaso, P., S. Moretti, I. Xenarios, M. Orobitg, A. Montanyola, J. M. Chang, J. F. Taly & C. Notredame, (2011) T-Coffee: a web server for the multiple sequence alignment of protein and RNA sequences using structural information and homology extension. *Nucleic Acids Res* **39**: W13-17.

Edgar, R. C., (2004) MUSCLE: multiple sequence alignment with high accuracy and high throughput.

Nucleic Acids Res **32**: 1792-1797.
Emsley, P. & K. Cowtan, (2004) Coot: model-building tools for molecular graphics. *Acta Crystallogr D Biol Crystallogr* **60**: 2126-2132.
Evans, P., (2006) Scaling and assessment of data quality. *Acta Crystallogr D Biol Crystallogr* **62**: 72-82.
Flyg, C., K. Kenne & H. G. Boman, (1980) Insect pathogenic properties of *Serratia marcescens*: phage-resistant mutants with a decreased resistance to *Cecropia* immunity and a decreased virulence to *Drosophila*. *J Gen Microbiol* **120**: 173-181.
Gasteiger, E., C. Hoogland, A. Gattiker, S. Duvaud, M. R. Wilkins, R. D. Appel & A. Bairoch, (2005) Protein identification and analysis tools on the ExPASy server. In: The Proteomics Protocols Handbook. pp. 571-607.
Grinter, N. J., (1983) A broad-host-range cloning vector transposable to various replicons. *Gene* **21**: 133-143.
Guzman, L. M., D. Belin, M. J. Carson & J. Beckwith, (1995) Tight regulation, modulation, and high-level expression by vectors containing the arabinose PBAD promoter. *J Bacteriol* **177**: 4121-4130.
Herrero, M., V. de Lorenzo & K. N. Timmis, (1990) Transposon vectors containing non-antibiotic resistance selection markers for cloning and stable chromosomal insertion of foreign genes in gram-negative bacteria. *J Bacteriol* **172**: 6557-6567.
Jack, R. L., G. Buchanan, A. Dubini, K. Hatzixanthis, T. Palmer & F. Sargent, (2004) Coordinating assembly and export of complex bacterial proteins. *EMBO J* **23**: 3962-3972.
Kabsch, W. & C. Sander, (1983) Dictionary of protein secondary structure: pattern recognition of hydrogen-bonded and geometrical features. *Biopolymers* **22**: 2577-2637.
Kaniga, K., I. Delor & G. R. Cornelis, (1991) A wide-host-range suicide vector for improving reverse genetics in gram-negative bacteria: inactivation of the *blaA* gene of *Yersinia enterocolitica*. *Gene* **109**: 137-141.
Krissinel, E. & K. Henrick, (2004) Secondary-structure matching (SSM), a new tool for fast protein structure alignment in three dimensions. *Acta Crystallogr D Biol Crystallogr* **60**: 2256-2268.
Krissinel, E. & K. Henrick, (2007) Inference of macromolecular assemblies from crystalline state. *J Mol Biol* **372**: 774-797.
Kurz, C. L., S. Chauvet, E. Andres, M. Aurouze, I. Vallet, G. P. Michel, M. Uh, J. Celli, A. Filloux, S. De Bentzmann, I. Steinmetz, J. A. Hoffmann, B. B. Finlay, J. P. Gorvel, D. Ferrandon & J. J. Ewbank, (2003) Virulence factors of the human opportunistic pathogen *Serratia marcescens* identified by *in vivo* screening. *EMBO J* **22**: 1451-1460.
Leslie, A. G., (2006) The integration of macromolecular diffraction data. *Acta Crystallogr D Biol Crystallogr* **62**: 48-57.
McCoy, A. J., R. W. Grosse-Kunstleve, P. D. Adams, M. D. Winn, L. C. Storoni & R. J. Read, (2007) Phaser crystallographic software. *J Appl Crystallogr* **40**: 658-674.
Murdoch, S. L., K. Trunk, G. English, M. J. Fritsch, E. Pourkarimi & S. J. Coulthurst, (2011) The opportunistic pathogen *Serratia marcescens* utilizes type VI secretion to target bacterial competitors. *J Bacteriol* **193**: 6057-6069.
Murshudov, G. N., P. Skubák, A. A. Lebedev, N. S. Pannu, R. A. Steiner, R. A. Nicholls, M. D. Winn, F. Long & A. A. Vagin, (2011) REFMAC5 for the refinement of macromolecular crystal structures. *Acta Crystallogr D Biol Crystallogr* **67**: 355-367.
Rao, V. A., S. M. Shepherd, G. English, S. J. Coulthurst & W. N. Hunter, (2011) The structure of *Serratia marcescens* Lip, a membrane-bound component of the type VI secretion system. *Acta Crystallogr D Biol Crystallogr* **67**: 1065-1072.
Terwilliger, T. C., (2003) Automated main-chain model building by template matching and iterative fragment extension. *Acta Crystallogr D Biol Crystallogr* **59**: 38-44.
Winn, M. D., C. C. Ballard, K. D. Cowtan, E. J. Dodson, P. Emsley, P. R. Evans, R. M. Keegan, E. B. Krissinel, A. G. W. Leslie, A. McCoy, S. J. McNicholas, G. N. Murshudov, N. S. Pannu, E. A. Potterton, H. R. Powell, R. J. Read, A. Vagin & K. S. Wilson, (2011) Overview of the CCP4 suite and current developments. *Acta Crystallogr D Biol Crystallogr* **67**: 235-242.
Winn, M. D., M. N. Isupov & G. N. Murshudov, (2001) Use of TLS parameters to model anisotropic displacements in macromolecular refinement. *Acta Crystallogr D Biol Crystallogr* **57**: 122-133.
